# Radiotherapy and chemotherapy change vessel tree geometry and metastatic spread in a small cell lung cancer xenograft mouse tumor model

**DOI:** 10.1371/journal.pone.0187144

**Published:** 2017-11-06

**Authors:** Thorsten Frenzel, Bertin Hoffmann, Rüdiger Schmitz, Anja Bethge, Udo Schumacher, Gero Wedemann

**Affiliations:** 1 Center for Experimental Medicine, Department of Anatomy and Experimental Morphology, University Cancer Center, University Medical Center Hamburg-Eppendorf, Hamburg, Germany; 2 University Hospital Hamburg-Eppendorf, Ambulatory Center, Department for Radiation Oncology, Hamburg, Germany; 3 Competence Center Bioinformatics, Institute for Applied Computer Science, University of Applied Sciences Stralsund, Stralsund, Germany; University of South Alabama Mitchell Cancer Institute, UNITED STATES

## Abstract

**Background:**

Tumor vasculature is critical for tumor growth, formation of distant metastases and efficiency of radio- and chemotherapy treatments. However, how the vasculature itself is affected during cancer treatment regarding to the metastatic behavior has not been thoroughly investigated. Therefore, the aim of this study was to analyze the influence of hypofractionated radiotherapy and cisplatin chemotherapy on vessel tree geometry and metastasis formation in a small cell lung cancer xenograft mouse tumor model to investigate the spread of malignant cells during different treatments modalities.

**Methods:**

The biological data gained during these experiments were fed into our previously developed computer model “Cancer and Treatment Simulation Tool” (CaTSiT) to model the growth of the primary tumor, its metastatic deposit and also the influence on different therapies. Furthermore, we performed quantitative histology analyses to verify our predictions in xenograft mouse tumor model.

**Results:**

According to the computer simulation the number of cells engrafting must vary considerably to explain the different weights of the primary tumor at the end of the experiment. Once a primary tumor is established, the fractal dimension of its vasculature correlates with the tumor size. Furthermore, the fractal dimension of the tumor vasculature changes during treatment, indicating that the therapy affects the blood vessels’ geometry. We corroborated these findings with a quantitative histological analysis showing that the blood vessel density is depleted during radiotherapy and cisplatin chemotherapy. The CaTSiT computer model reveals that chemotherapy influences the tumor’s therapeutic susceptibility and its metastatic spreading behavior.

**Conclusion:**

Using a system biological approach in combination with xenograft models and computer simulations revealed that the usage of chemotherapy and radiation therapy determines the spreading behavior by changing the blood vessel geometry of the primary tumor.

## Introduction

The formation of distant metastases is the leading cause of death in cancer patients [1]. Metastasis formation is a complex process that already begins when the first malignant cell starts to divide [2–4]. Once the growing tumor has reached a certain size, it sends out angiogenic signals so that blood vessels start growing into the tumor. Future metastatic cells then detach from the primary tumor mass and invade the blood vessel in a process known as intravasation. To form metastases these cells must survive in the circulation and attach to the endothelium at the site of the future metastasis. After transmigration of the endothelium, the cancer cell lodges in the connective tissue of the host organ and starts to divide again, thus creating a vicious circle of metastasis formation.

Blood vessels play important roles in many aspects of malignant progression. They provide channels for the distribution of oxygen and nutrients to cells which is crucial for the growth of the primary tumor. In normal tissue, the vasculature exhibits a hierarchical structure with respect to the blood vessel radius and length. In contrast to normal tissues, the vessel tree geometry in primary tumors is disorganized and hence more variable in the order of branches and less hierarchical [5]. The tumor blood vessels provide channels for the dissemination of malignant cells, hence it is reasonable to assume that the geometry of the tumor vasculature influences the number of cells that undergo intravasation and eventually form metastases [6,7]. Attempts have been made to pharmacologically inhibit the growth of blood vessels in tumors but these antiangiogenic therapies impair the transport not only of oxygen and nutrients but also of vital therapeutic agents [8]. The poor blood vessel structures result in hypoxia and this also influences the effectiveness of both radiation and chemotherapy [7,9]. In addition, it may also modulate the infiltration of immune competent cells into the tumor and thus influences immune response. Thus, a selection pressure is generated on the tumor cells towards a more malignant genotypes [10].

Both disease progression and treatment response therefore closely depend on the geometry of the tumor vasculature and vice versa. A thorough understanding of these interdependences and interactions may aid the design of new therapeutic approaches that combine radiotherapy, antiangiogenic therapy and conventional chemotherapy [10–12]. The stronger the reciprocal dependence of vascular geometry and therapeutic effect, the more important it is that a thorough understanding of this interdependency can be established.

It has been suggested that cancer therapies do not only reduce the mass of the primary tumor but also influence the blood vessel geometry [13,14]. Whether and how this changed blood vessel geometry influences the metastatic behavior is unclear so far. In order to clarify the impact of changes of the geometry of the tumor vasculature by different treatment interventions on the metastatic behavior we studied experimental data from xenograft mouse tumor models. We analyzed the data with our previously developed computer model CaTSiT [15,16] to investigate the metastatic behavior. We identified changes of the metastatic behavior and connected them to alterations of the global vessel tree geometry and arrangement in terms of vessel density and fractal dimension in the primary tumor during cisplatin chemotherapy and hypofractionated radiotherapy. These findings were corroborated by the results of a subsequent quantitative histological analysis of the primary tumors. Furthermore, our approach confirms a relationship between the tumor size and geometry of its vasculature. Thus chemotherapy and radiotherapy not only change the mass of the primary tumor tissue but also modifies the metastatic behavior by altering the blood vessel geometry. This finding opens a new starting point for the development of new therapeutic intervention strategies for the treatment of distant metastasis formation.

## Materials and methods

In this article we analyze data from a xenograft mouse tumor model. OH1 small cell lung cancer (SCLC) cells we injected subcutaneously into the flank of the mice to form a local tumor nodule. One group of mice was treated with chemotherapy (ChT), another received radiotherapy (RT), and a third group had no therapy (E). During local development of a local tumor nodule the size of tumors was measured repeatedly. At the end of the experiment the animals were sacrificed and the metastatic cell load in the lungs was determined. Data from the untreated group was used to study the growth and spreading behavior without therapy. Data from the treated groups were used to investigate the impact of chemotherapy or radiotherapy with our computer model. Inspired by the results from the simulations we analyzed the blood vessel densities applying quantitative histology.

### Animal experiments and histological preparation

All animal experiments were approved by the local animal experimental approval committee (permission ID G14/13, Freie und Hansestadt Hamburg, Behörde für Gesundheit und Verbraucherschutz). They are in accordance with the relevant national and international guidelines. This article analyzes existing data from a larger experiment (submitted for publication by Frenzel et al.) so that no additional animals had to be sacrificed. The following paragraphs summarize the important details needed for our data analysis.

1x10^6^ human OH1 small cell lung cancer (SCLC) cells were subcutaneously injected in a total volume of 200 μl at the right lower trunk of severe combined immunodeficient (SCID) mice (CB17/Icr-Prkdc^scid^/IcrIcoCrl from Charles River Laboratories International Inc., Wilmington, MA, USA) to form a local primary tumor. The OH1 tumor cells were injected with matrigel mixed 1:2 with cell culture medium without further additives [17]. The established SCLC cell line OH1 was generously provided by Dr. U. Zangemeister-Wittke, Bern, Switzerland and was initially described by Gazdar et al. [18]. The cells were checked for mycoplasma by PCR-based VenorGeM Mycoplasma Detection Kit (Minerva Biolabs GmbH, Berlin, Germany).

The tumor cells formed a palpable local tumor nodule within 14 to 21 days so that the experiment could start. The local tumor growth was measured by manual palpation in comparison with spheres of defined volumes. After this variable time period, the animals were assigned to one of three subgroups: i) the radiotherapy group (RT); ii) the chemotherapy group (ChT); and iii) the control group (E), demonstrating tumor growth without therapy. As we were limited in the number of animals that we could treat per day, we repeated the experiment twice to have at least 5 animals that could be evaluated for our analysis.

Equivalent to locally ablative radiotherapy in humans, RT was administered in five fractions of 10 Gy on consecutive days with an X-ray tube (Rich. Seifert & Co. GmbH & Co. KG, Ahrensburg, Germany) [19]. ChT was applied as an intraperitoneal injection of cisplatin (6 mg/kg) on the same day as radiotherapy commenced. Mice were sacrificed 14 days after the start of therapy (RT, ChT, E). The local primary tumors were removed and embedded in paraffin for further histological analysis and tissue samples of the lung, liver, and brain were collected for ALU-PCR and conventional wax histology. Blood and bone marrow samples were also analyzed by ALU-PCR.

For determining the density of blood vessels, three sections, each of them 100 μm apart, were cut from each paraffin-embedded primary tumor. Endothelial cells were stained immunohistochemically by use of an anti-CD31 monoclonal antibody and visualization was achieved by an avidin-biotin alkaline phosphatase complex. In addition, double labeling immunohistochemistry was performed on the primary tumors for detecting endothelial and apoptotic cells. Endothelial cells were detected using an anti-CD31 monoclonal antibody and an avidin-biotin horseradish peroxidase complex. Apoptotic and cells with activated DNA damage pathway were stained using an anti-histone H2AX monoclonal antibody and an avidin-biotin alkaline phosphatase complex. Conventional and double labeling immunohistochemistry have successfully been applied to 10, 9 and 7 tumors for E, ChT and RT groups, respectively. Histological slides were digitalized using a Mirax Midi scanner (Carl Zeiss, Oberkochen, Germany). For details refer to the supporting methods text, section A.

Tumor cells in distant organs (circulating tumor cells: CTCs; disseminated tumor cells: DTCs; micrometastases) were measured by ALU-PCR as described below. In order to validate the presence of metastases eight paraffin-embedded lung sections from one animal were stained by a human-specific anti-NCAM antibody (clone 301021, R&D Systems, Inc., Minneapolis, USA).

### Image segmentation

The data of the scanned slides were analyzed by custom-made software using Matlab (Matlab R2015b, The MathWorks Inc., Natick, USA). Candidate pixels were identified in {L,a,b} color space and used as seeds for a region growing approach to segment the blood vessels from the histological slide [20,21]. The resulting figures for the blood vessel densities were created using the toolbox notBoxPlot [22]. For comparison, another segmentation algorithm based on the k-means clustering method was implemented with Matlab and the Matlab Statistics and Machine Learning Toolbox [21,23]. Further details are described in section B in the S1 Text and S1 Fig.

### Detection of circulating and disseminated tumor cells through qPCR

For each mouse, the left lung, a part of the liver and brain tissue were homogenized in the sample disruptor TissueLyser II (Qiagen GmbH, Düsseldorf, Germany) and the DNA isolated using the QIAamp DNA Mini Kit (Qiagen) according to the manufacturer’s protocol. Bone marrow was collected by flushing the left femora with 1 ml NaCl 0.9%. 200 μl of blood and the bone marrow suspensions were subjected to DNA isolation using the QIAamp DNA Blood Mini Kit (Qiagen). Quantitative Alu PCR was applied to search for circulating tumor cells (CTCs) in the blood and disseminated tumor cells (DTCs) in lung, liver, bone marrow, and brain [24–26]. Tissue samples were normalized to 30 ng/μl DNA and 2 μl of this solution was used as a template. Numbers of CTCs in this study are given per 20 ng template and numbers of DTCs per 60 ng template, which corresponds to approximately 10,000 murine cells. The absolute number of disseminated tumor cells was calculated as described in section C in the S1 Text.

### Computer simulation model

We utilized our previously developed computer model CaTSiT [15,16,27] based on the mathematical model by Iwata et al. [6] to perform computer simulations of the spread of the SCLC cells.

A description of the simulation model can be found in the section D in the S1 Text, S2 Fig and also in [16] and technical details in [15]. The simulation software and some configuration files from published articles are freely available as open source [28]. The most important parameters for the simulation setup are given in S1 Table, whereby most of the parameters were determined by analyzing experimental data.

### Determining growth parameters of the primary tumor

**Fig 1:**
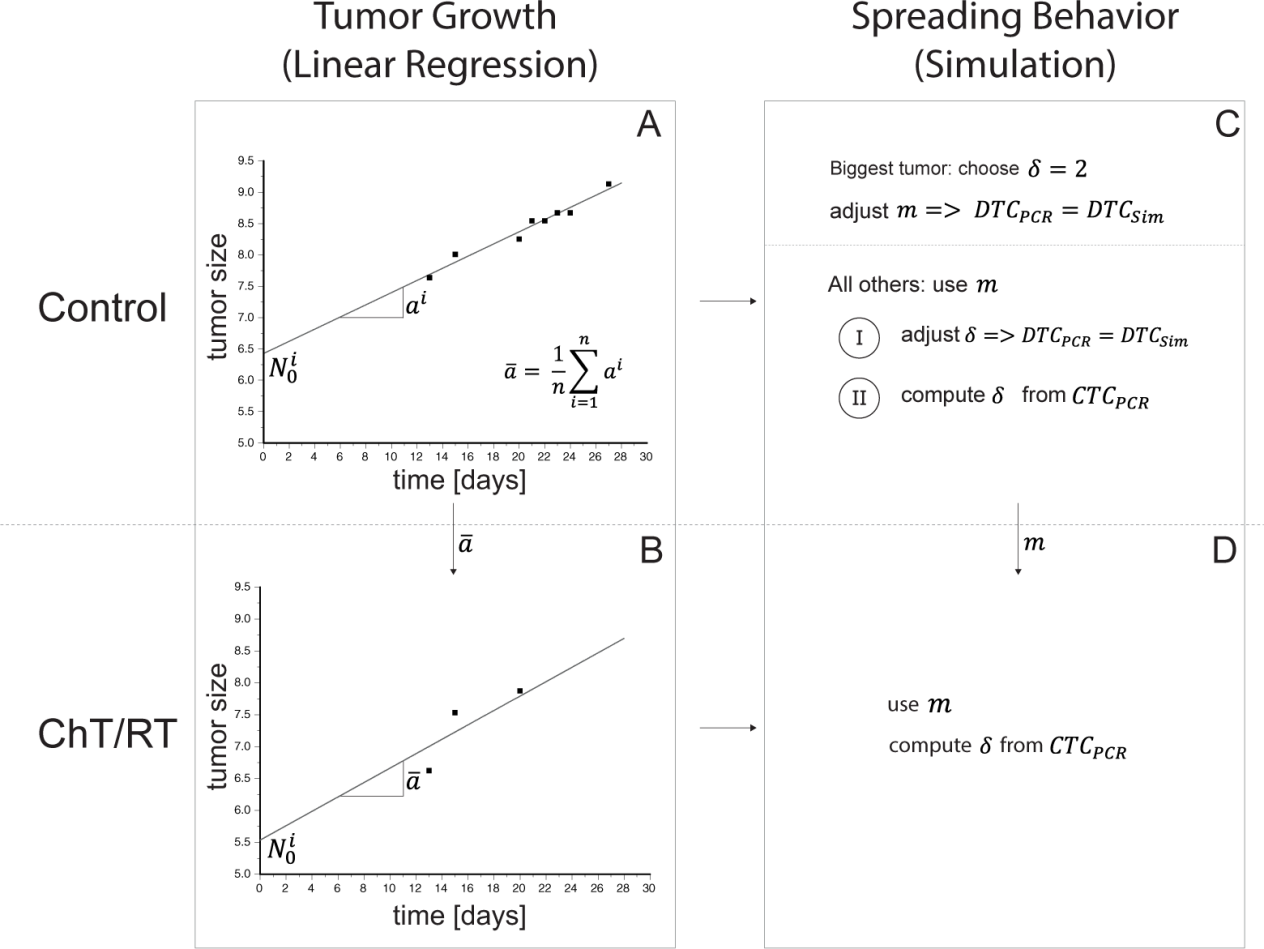
Overview of the workflow used to analyze the experimental data. Panel A shows the linear regression procedure to determine the number of engrafted tumor cells $N_{0}^{i}$ and the growth rate constant *a*^*i*^ for each mouse i from the untreated tumor bearing mice of the control group. In Panel B, the mean growth rate constant $\bar{a}$ from the animals of this control group was used to determine the number of engrafted tumor cells in the groups of animals that were treated with radiotherapy or chemotherapy by using only the data points which were unaffected by therapy because we need a parametrization of the normal growth behavior of the tumor since the therapy effect will be modelled separately. Using this approach, the effects of the therapy do not affect the parametrization of the growth function. Panel C shows the approach to determine the spreading behavior in the control group, which is represented by the colonization coefficient *m* and the fractal dimension *δ*. Parameters were determined in two different ways: (I) from the experimentally determined numbers of disseminated tumor cells (DTC) and (II) from the experimentally numbers of circulating tumor cells (CTC). For the mouse with the biggest tumor we choose a fractal dimension of 2, which implies a blood supply on the surface of the tumor, to determine the colonization coefficient. Afterwards, we calculated with these data all other parameters for the remaining mice in the control group. In Panel D, the determined parameter m will be used to compute the fractal dimension *δ* in treated groups with its own growth parameters which were determined in panel B.

The number of cells *x*(*t*) of the primary tumor is modeled by the exponential function

$$x(t)=N_{0}e^{at},$$ (1)

where *N*_0_ is the number of engrafted tumor cells at time *t* = 0 and *a* is the growth rate constant. The often used sigmoidal Gompertz growth function or the spheroid model both were not modelling the experimental data (see section E in the S1 Text and S4 Fig) [29]. The parameters for the growth rate constant *a* and the number of engrafted tumor cells *N*_0_ in Eq (1) were determined by using linear regression (Fig 1A and 1B, section F in the S1 Text and S5 Fig).

### Modeling the spread of malignant cells

The number of cells that spread from the primary tumor per time unit is described by a colonization rate β(x) in the model of Iwata et al. [6]. Adopting the argument given therein, the colonization rate can be written as

$$\beta (x)=mx^{\delta /3},$$ (2)

where m is the colonization coefficient and δ is the fractal dimension of blood vessels [29]. The fractal dimension describes the blood vessel geometry and, thus, the nutrient supply of the primary tumor or metastases, respectively. The fractal dimension can vary in an interval from 0 to 3. A fractal dimension δ of 2 implies that nutrients are supplied only at the surface of the tumor whereas a fractal dimension δ of 3 implies that nutrients are delivered throughout the tumor (Hoffmann et al, in Press). Even small changes of the fractal dimension may have a large effect on the number of cells which were spread from the primary tumor (see S1 Text and S3 Fig). We used two different methods to determine these parameters, one using the measured disseminated tumor cells (*DTC*_*PCR*_) in the lung and one using the circulating tumor cells (*CTC*_*PCR*_) (Fig 1C and 1D, section F in the S1 Text). Both were measured by ALU-PCR.

### Modeling the treatment groups

In the RT and ChT groups, the size of the primary tumor is affected by the respective treatment over time. Therefore, we could not determine the growth and spreading behavior as described for the control group, because data points that were affected by treatment falsify the determined growth parameter by using linear regression (section F in the S1 Text). As the same cell line was implanted into all the mice, we used the previously determined mean growth rate constant $\bar{a}$ and colonization coefficient *m* from the control group. A detailed description of the modeling process and the determined parameters of the chemo- and radiotherapy are given in section F in the S1 Text, S6 and S7 Figs and also in S3 and S4 Tables.

## Results

### Variation in the numbers of engrafted cells

**Fig 2:**
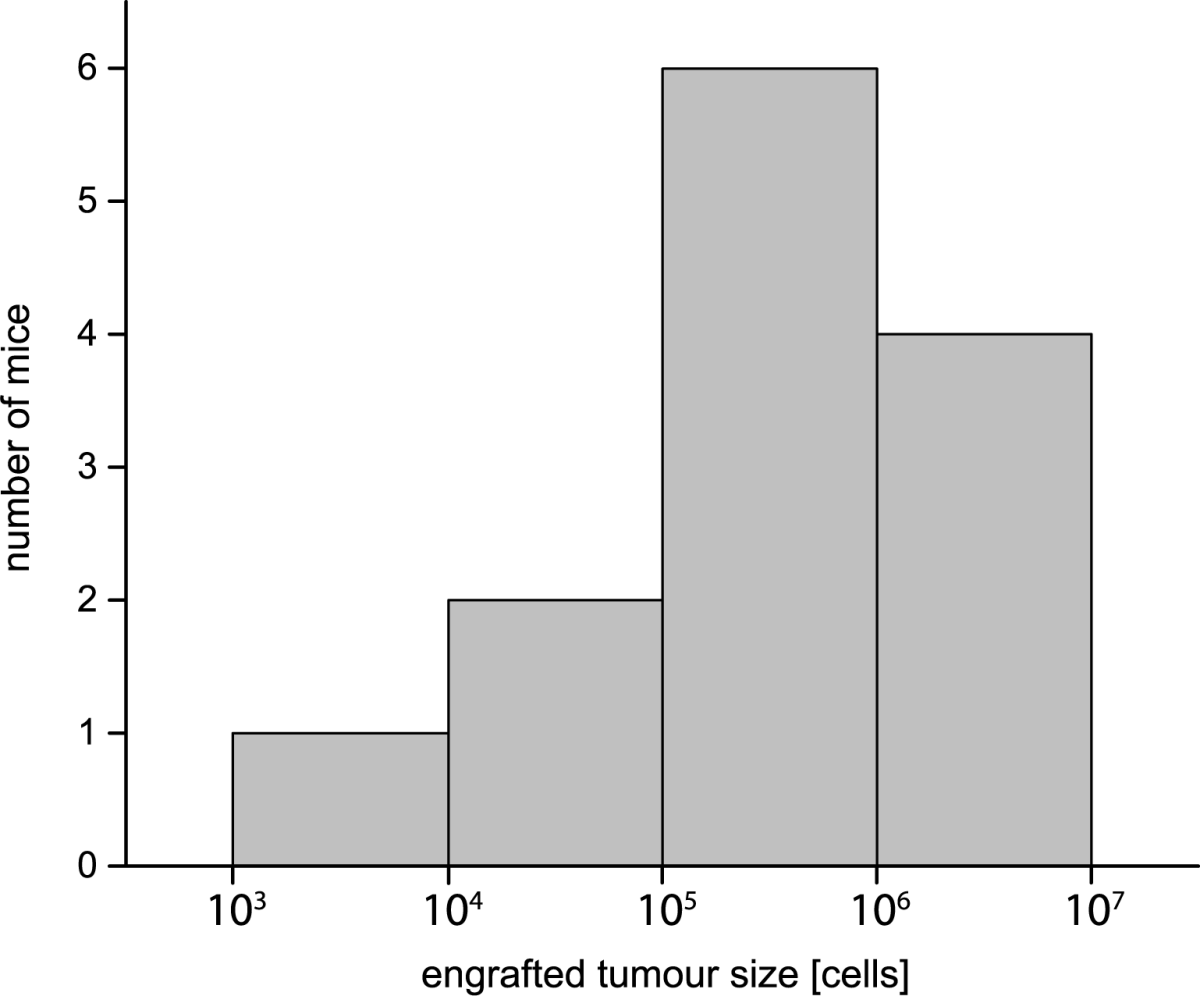
Histogram showing the number of engrafted tumor cells N_0_ in the control group at day 0.

SCLC cells were subcutaneously injected into immunodeficient SCID mice to form a local primary tumor. During growth, the size of each tumor was measured by palpation at different time steps. In addition, each tumor was weighted at necropsy. The latter measurement being more precise than palpation, the difference of measured tumor weight and palpated tumor size was used to correct for systematic errors in the palpated growth curves (ref. section F in the S1 Text). The S5–S7 Figs show the corrected measurements for the tumor size growth of the animals in the control, ChT and RT groups. The measured development of the tumor sizes was fitted by linear regression to yield the parameters *N*_0_^*i*^ and *a*_*i*_ (see section F in the S1 Text and S5 and S7 Figs). We calculated a mean growth rate constant *a* of 0.260 ± 0.043 day^−1^ for the control group. The parameters for the number of engrafted tumor cells *N*_0_ were determined for each mouse individually in this group. The mean *N*_0_ value was calculated as (1.44 ± 2.37) × 10^6^ which corresponds well with the number of 1 x 10^6^ injected tumor cells. The distribution of the values is presented as histogram in Fig 2 and the values for all mice in the control group are given in S2 Table. For treated groups, parameters can be found in S3 and S4 Tables. Please note that the histogram in Fig 2 does not represent a Gaussian distribution. Therefore, the standard derivation is greater than the computed mean value of the engrafted tumor cells *N*_0_.

### The geometry of the tumor vasculature changes with tumor size

**Fig 3:**
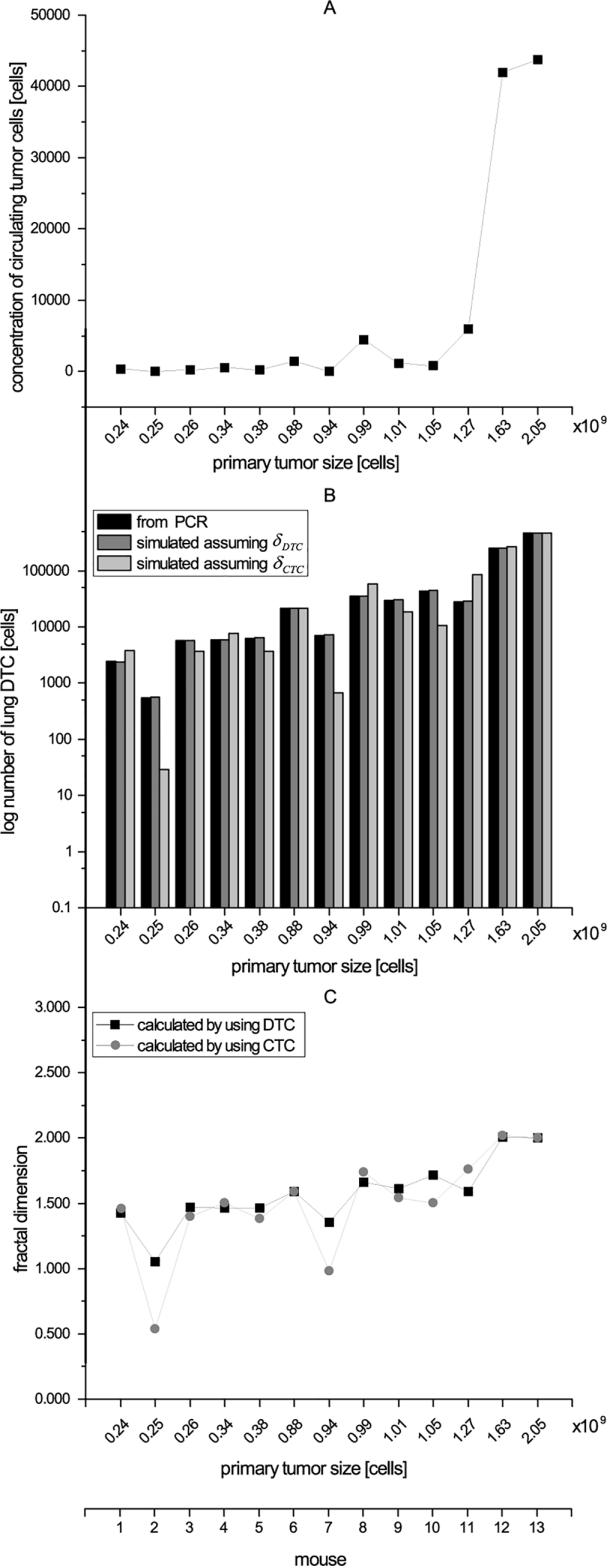
The effect of tumor size on tumor vasculature. Panel A shows the relationship between the size of the primary tumor and the concentration of CTC. In panel B the numbers of disseminated tumor cells, which were determined by PCR, and the calculated number of disseminated tumor cells using two evaluation methods are compared: Fractal dimension (DTC)—the fractal dimension *δ*_*DTC*_ was adjusted until the number of *DTC*_*Sim*_ reached the number of *DTC*_*PCR*_. Fractal dimension (CTC)—the fractal dimension *δ*_*CTC*_ was computed based on the number of circulating tumor cells. Panel C compares the fractal dimension based on both evaluation methods. In all panels, the values are sorted by the size of the primary tumor in ascending order (x-axis primary tumor size [cells]).

In order to investigate the relationship between vasculature and tumor size, we weighed the primary tumor at the end of the experiment and determined the number of CTCs by ALU-PCR measurements. Fig 3A shows the dependency of the concentration of CTCs on the size of the primary tumor. These data were compared with the results from the computer simulations regarding the blood supply of the primary tumor, which is indicated by the fractal dimension (Fig 3B). The experimental data indicates that generally, the greater the size of the primary tumor, the higher the number of CTCs (Fig 3A). In addition, the fractal dimension determined by simulation shows that a larger primary tumor has a better vascularization than a smaller one, which means the larger primary tumor gets more nutrients (Fig 3C). The Pearson correlation coefficient shows a positive linear correlation (r = 0.8059805) between the size of the primary tumor and the number of CTCs. Statistical analysis was performed by using R 3.4.0.

### The total number of circulating tumor cells provides information about the metastatic behavior

**Fig 4:**
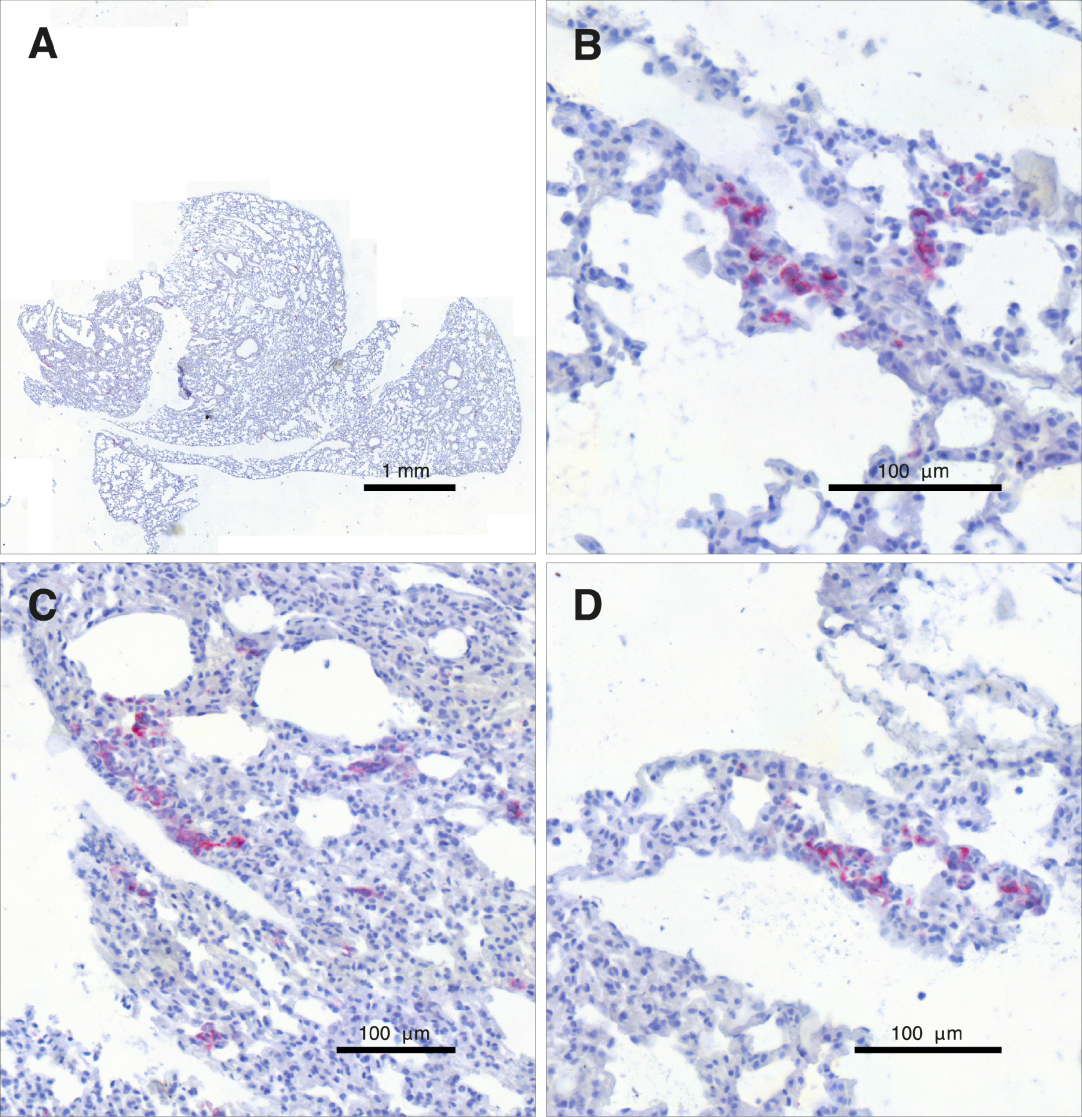
Human-specific anti-NCAM staining revealed the existence of numerous clusters of malignant SCLC cells in the animal’s lung. An overview of a lung section with the metastatic cells stained red is shown in panel A. The panels B–D provide detailed views of the same slide and show several DTC clusters inside the animal’s lung.

Use of CaTSiT and the underlying mathematical model by Iwata et al. [6] require the sensitive measurement of disseminated tumor cells (DTCs) in distant organs. In this paper, ALU-PCR is used as a sensitive means of measuring the concentration of human cells in these organs. To validate this molecular approach, Fig 4 shows an N-CAM stained lung (A) and a number of detailed views of the same section (B–D). Several clusters of DTCs validate micrometastases so that the ALU-PCR really measured metastases and not only CTCs.

**Fig 5:**
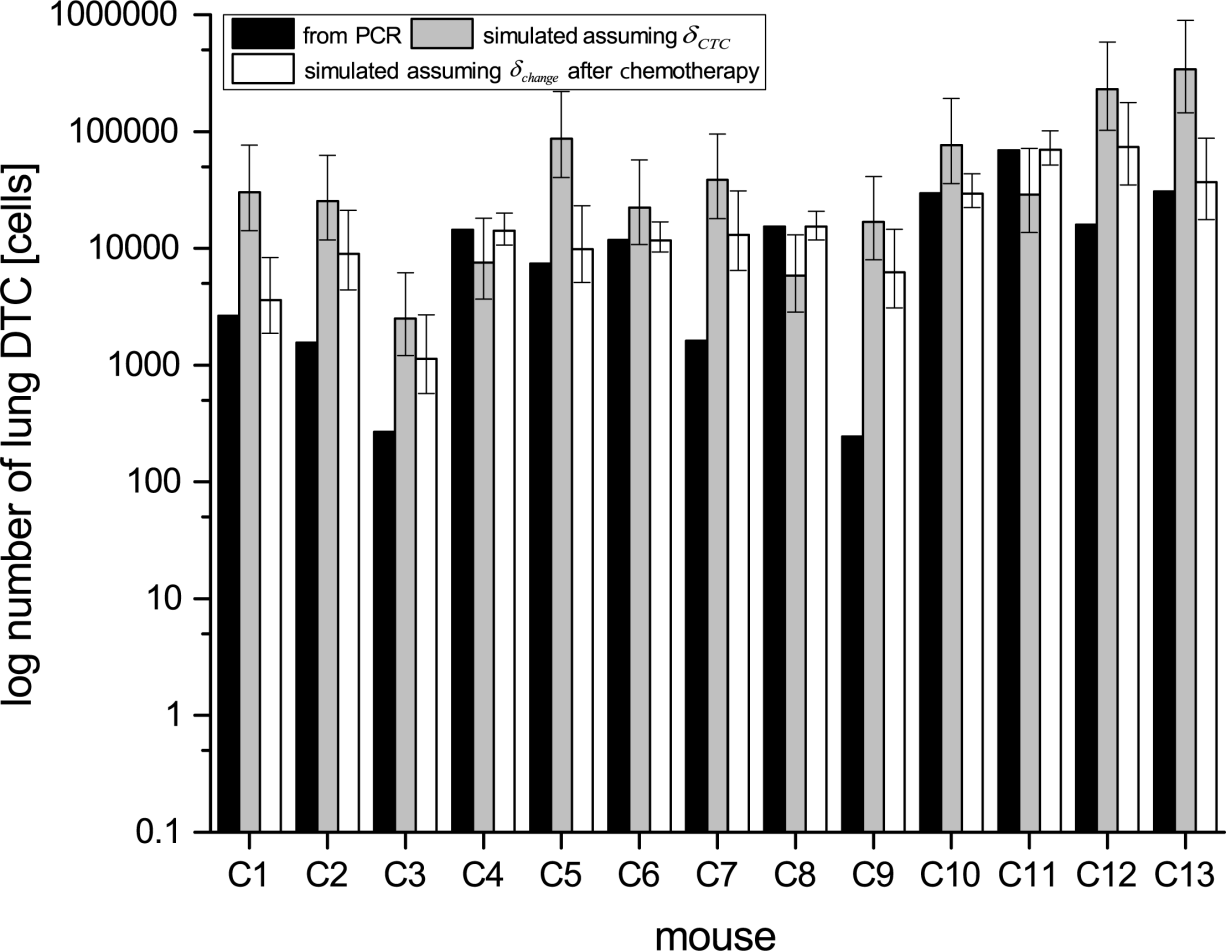
Number of disseminated tumor cells (DTCs) in the lung of mice in the chemotherapy group measured by PCR (black bars) compared with results from simulations with computed fractal dimension (grey bars) and adjusted fractal dimension (white bars). Simulation results with adjusted fractal dimension fit better to experimental data. Asterisks indicate mice, where simulations with adjusted fractal dimension are in the range of the statistical error with the measured values.

Note that, for each treatment group, the experimental results for CTC and DTC concentrations in the blood and the peripheral organs as measured by ALU-PCR are given in the figures in which also the results of CaTSiT modeling and analysis for the same group are shown, namely Fig 3A and 3B (control group) as well as Fig 5 (ChT).

The dependence of the metastatic behavior of the primary tumor on its size was modelled with the colonization rate *β(x)* (Eq (2)). A reference mouse was selected from the control group that had the largest primary tumor at the end of the experiment. After including the numbers of DTCs determined by Alu PCR (*DTC*_*PCR*_), the colonization coefficient *m* was calculated to be 50 × 10^−3^ (cell × day)^−1^.

The fractal dimensions *δ* of the blood vessels in the primary tumor were determined for each mouse in two different ways: (A) by adjusting *δ* so that the number of DTCs fits to the experimental data (*δ*_*DTC*_) and (B) from the measurements of the CTCs (*δ*_*CTC*_). Fig 3C shows that the results of both methods fit well. Only mice 2 and 7 showed deviations because of their low number of CTC. To cross-check both approaches, we compared the number of DTCs computed with both methods. Fig 3B shows that both methods gave similar results, indicating that the number of CTCs can be used to compute the parametrization of the spreading behavior. The Wilcoxon Signed-Rank test fails to reject the null hypothesis that the computed fractal dimension in both ways (*δ*_*DTC*_, *δ*_*CTC*_) are similar (p = 0.2896). This result supports our hypothesis that the number of circulating tumor cells in the blood stream can be also used to compute the fractal dimension of the primary tumor and not only by the number of disseminated tumor cells. Statistical analysis was performed by using R 3.4.0.

The relationship between circulating tumor cells and metastatic behavior was shown for the untreated group. We used this relationship for simulation of the treated groups. It might be speculated, that this relation changes with treatments. Since there are no real indications for this, we refrained from including it to our model.

### Chemotherapy affects the vessel tree geometry of the primary tumor

Our computer simulation aimed to calculate the numbers of DTC in the lung also for animals that underwent ChT. Our results were in remarkably good agreement with results measured by ALU-PCR but could not reproduce the results for all of the animals. This observation indicates that not all effects occurring in the animals were taken into account by our calculation model. It has been suggested, that chemotherapy changes the fractal dimension of the blood vessels [13]. To test this hypothesis, we extended our simulation software to take these changes into account. We adjust delta until the simulation results are equal to the number of *DTC*_*PCR*_. The simulation results (*DTC*_*Sim*_) then predicted the number of DTCs determined by Alu PCR (*DTC*_*PCR*_) in 8 out of 13 cases (S3 Table and Fig 5, marked with asterisks). The Wilcoxon Signed-Rank test rejects the null hypothesis that the simulated results produced the same results as the results by additionally changing fractal dimension (p = 0.02661). This result supports our research hypothesis that the fractal dimension decrease in most of the cases during the application of chemotherapy. Statistical analysis was performed by using R 3.4.0.

The single-dose chemotherapy therefore has a considerable effect on the blood vessel geometry, but a minor effect on the primary tumor volume itself. In cases where the fractal dimension were determined to *δ* = 0, this even implies a stop of the dissemination process. These findings are also reflected in the therapy-induced depletion of the vascular density, as discussed in the next section.

Assuming an altered fractal dimension from day one after the chemotherapy until the end of the experiment, the simulation results of the number of DTCs was as high as the number of DTCs determined by PCR in the range of the statistical error in 8 out of 13 cases. In order to estimate the statistical error for each mouse, error bars were calculated by simulating each mouse with all parameters with its determined value from regression and simulation, but in every complete simulation run of each mouse only one parameter differs to its maximum or minimum with respect to the parameters standard deviation. Finally, all possible results were computed following propagation of error. At a 0.05 significance level, we conclude that the results with an altered fractal dimension (white bar) are significantly better than the results without these adjustments (gray bar).

### Hypofractionated radiotherapy and cisplatin chemotherapy impair the tumor vasculature

The above simulation results strongly suggest fundamental changes to the tumor vasculature through ChT and RT. For this reason, we directly examined changes to the vessel tree through a histopathological study of the blood vessel densities in the three different treatment groups. To this end, we applied a region growing-based segmentation algorithm (see section B in the S1 Text, steps A1-A7, and S1 Fig) to sections cutfrom the primary tumors (3 sections per animal) with the blood vessels stained immunohistochemically (cf. Materials & Methods as well as section A in the S1 Text).

**Fig 6:**
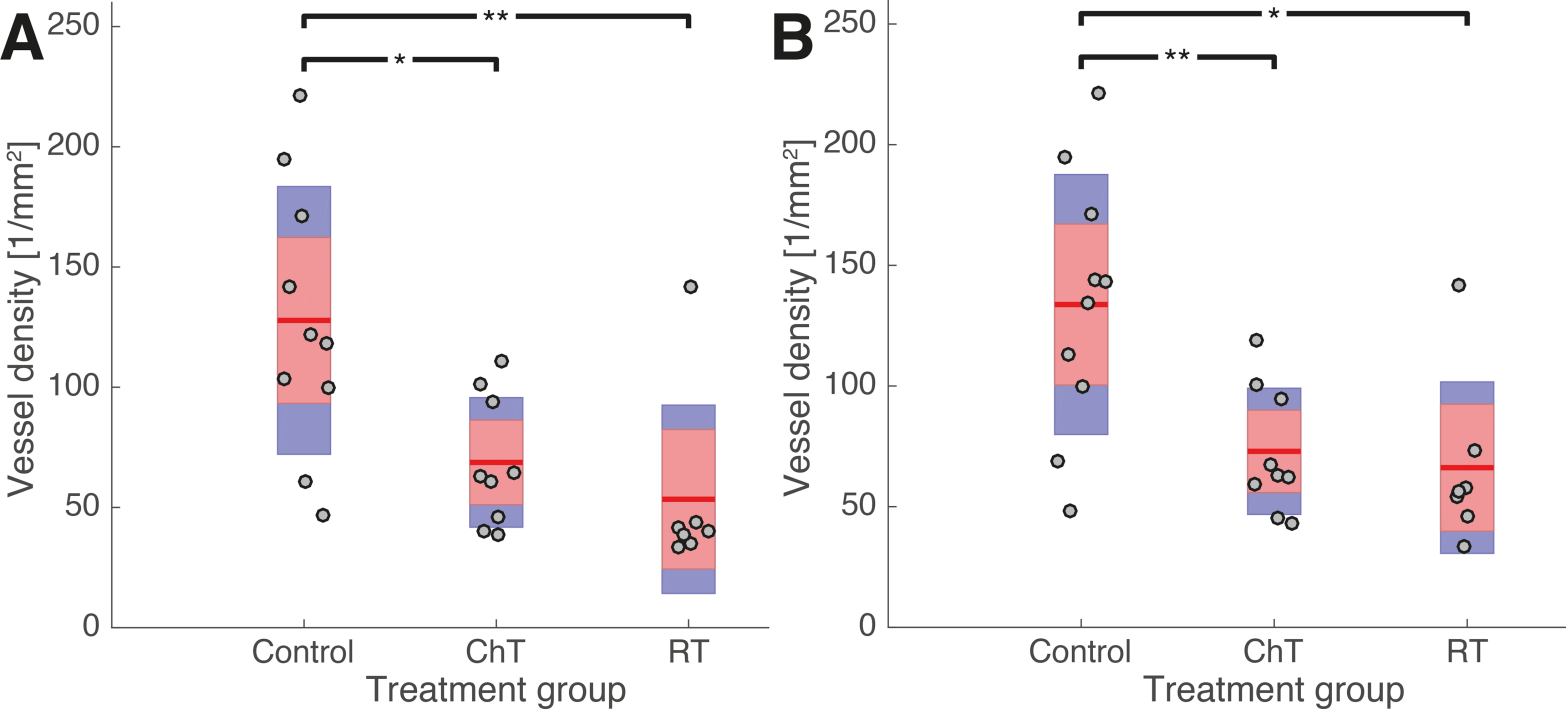
Blood vessel densities in untreated tumors (control group) and tumors that have been treated by cisplatin (ChT) and hypofractionated radiotherapy (RT). The blood vessel densities are computed with respect to (A) the entire tumor, and (B) only to the viable and homogeneous tumor regions, i.e. excluding areas of necrosis. In both cases, the blood vessel counting was not restricted to certain fields of view but include the whole region under consideration (cf. section B in the S1 Text). The margins of the tumors were excluded from the blood vessel counting due to unspecific staining this region. Each grey dot indicates the vessel density for a single tumor, which had been computed as the arithmetic mean over a number of histological sections (cf. section B of the S1 Text). The arithmetic mean of the entire treatment group (Control, ChT, RT) is shown by a red line. Red boxes indicate one standard deviation of the vessel densities in the respective group. The blue boxes indicate the 95% confidence intervals of the arithmetic mean values. An asterisk represents a significance level of p<0.05, two asterisks denote a significance level of p<0.01.

**Fig 7:**
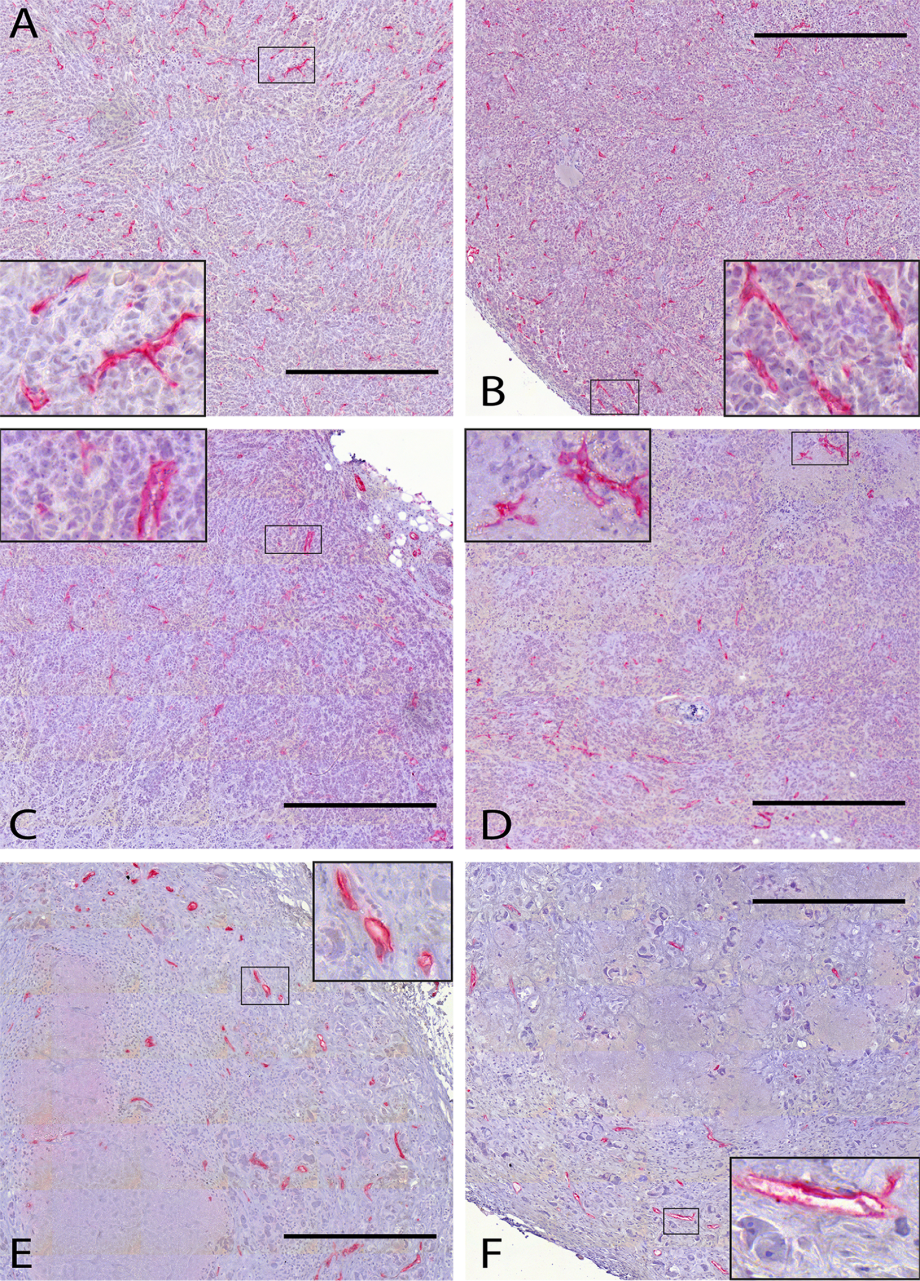
Histological sections from untreated tumors and tumors subjected to ChT or RT. In histological sections from the control (A, B), the ChT (C, D) and the RT group (E, F), the blood vessels are labeled red by immunohistochemical staining against CD31. Nuclei are counterstained by hematoxylin. The inset to each panel gives a detailed view of a smaller region outlined therein. All scale bars are 500 μm.

Fig 6 shows the vessel densities in the tumors of the control and the different treatment groups 15 days after the start of the respective treatment. The mice of the control group, which were not subjected to any treatment, were sacrificed at the same time. Fig 7 shows some histological sections of these groups with the blood vessels stained red.

As shown in Fig 6A, the arithmetic mean of the vessel density in the cisplatin (68.7/*mm*^2^) and radiotherapy groups (53.4/*mm*^2^) and also their median values (63.3/*mm*^2^ and 39.8/*mm*^2^, respectively) are outside one standard deviation around the mean of the control group corresponding to the interval of (119.8 ± 55.7)/*mm*^2^, and outside the 95% confidence interval for the arithmetic mean value of the control group vessel density. The blood vessel densities of six out of nine cisplatin-treated tumors and six out of seven RT-treated tumors lie outside this interval. Accordingly, the two-sample t-test rejects the null hypotheses that the vessel densities of the treatment groups and of the control group derive from the same normal distribution at the significance levels of p = 0.010 for ChT and p = 0.008 for RT.

Many tumors–particularly those that are large–are necrotic in their centers. These regions are notably different from the viable tumor regions around them. The necrotic central regions have a much thinner vasculature, if any. Even though their exact roles in the spawning of metastases are a topic current research, it is reasonable to assume that viable and necrotic regions play different roles in the metastatic progression. Likewise, necrotic and viable regions might also react differently to treatments. Therefore, one should differentiate between viable and necrotic tumor regions in a second step and study the vessel density and its changes in each of them separately.

Therefore, Fig 7B shows the vessel densities in the same tumors as Fig 6A, but restricted to their viable homogeneous regions (with the necrotic regions excluded by visual inspection). Still, the mean vessel densities of both the cisplatin (72.9/*mm*^2^) and radiotherapy group (66.2/*mm*^2^) and their median values (63.3/*mm*^2^ and 56.0/*mm*^2^, respectively) are outside one standard deviation of the control group mean, which corresponds to (133.8 ± 53.8)/*mm*^2^. The same six tumors from the ChT group and the same six tumors from the RT group, which we had found to lie outside one standard deviation around the control group average when considering the entire tumor samples, still do so upon restriction of the analysis to their vital regions. Again, the 95% confidence intervals of the mean values for both treatment groups do not include the arithmetic mean value of the control group, and vice versa, and the two-sample t-test again rejects the null hypothesis (p = 0.07 for cisplatin treatment and p = 0.011 for RT). Results for the necrotic regions alone are not statistically significant (data not shown).

These findings show that Cisplatin treatment and hypofractionated radiotherapy lead to a reduced vessel density in the entire tumor and in its viable regions alone, which is even detectable 15 days after the end of chemotherapy and eleven days after the last irradiation dose, respectively. Song et al. [14] recently showed that single-dose irradiation with 15 Gy can lead to a “transient collapse” of the blood vessels, followed by a considerable recovery of the vessel density from day 0 to day 6 post-treatment. In the light of this dynamic adaptation, we think that our findings of a significantly reduced vessel density 15 and 11 days after treatment might point to a long lasting or even permanent depletion of the tumor vasculature.

## Discussion

### General problems in mathematical simulation of the metastatic spread of tumor cells

The formation of distant metastases is a multi-step process that is not yet understood in all details. Small animal experiments can help to improve our knowledge of this process. As animal experiments must be limited by law to as few animals as possible and as detailed information needed for later computer simulations is not available for most experiments, there are only few experimental data available for computer simulation models. Our simulation software CaTSiT was developed and improved in recent years to model tumor growth, metastasis formation, and effects of different treatments like ChT and RT even when only limited data are available. Despite these restrictions assumptions have to be made to calculate the numbers of CTCs and DTCs with mathematical formulas e.g. the drug decay rate or the fraction of cells in the S phase.

### Changes of the fractal dimension and of the density of the blood vessels

Data from the ChT cohort revealed significant differences in the number of DTCs detected by PCR (*DTC*_*PCR*_) and the computer simulation (*DTC*_*Sim*_), although the simulation was performed with the same procedure regarding to the tumor growth and spreading behavior for the ChT group as for the control group. A possible explanation for this discrepancy is that the ChT alters the geometry of the blood vessel tree and consecutively changes the accessibility of the blood vessels for malignant cells. Once this access problem was taken into account, the data from the simulations agreed well with the experimental data. Therapy-induced changes of the fractal dimension δ and the density of the blood vessels are in agreement with our other studies, e.g. [13,14]. The treatment might also change the colonization coefficient *m*. Due to the setup of our approach it is not possible to distinguish between changes in δ and *m*. In order to clarify the proportion of changes in both parameters, more detailed experimental data are necessary. The study by Song et al. [14] emphasizes the importance of studying the detailed kinetics in order to fully understand therapy-induced changes to the tumor vasculature. The present study implies that also long-term effects (at least 15 days) need to be taken into account. This finding needs to be investigated further. It could be hypothesized, that the organ tissue in the distant lung changed during the chemotherapy treatment, which leads to a reduced ability to engraft tumor cells in the lung. But this possibility is speculative. Due to the alteration, which was confirmed by the histologically analyses and the results by other groups, we assume that the biggest influence is the change of the fractal dimension and the density of blood vessels of the primary tumor during treatment.

### Correlation between the size of the primary tumor and its blood supply is still unclear

The computational analysis of the experiments shows that the size of the primary tumor and its blood supply are correlated (Fig 3). However, with our approach it is not possible to determine whether (A) a tumor grows faster since it is well supplied with blood, or (B) larger tumors have more blood vessels for the supply of nutrients, or even both. Compared with the results from the control group, the reference mouse that had the greatest tumor size at the end of the experiment did not have the highest number of engrafting tumor cells at the beginning (day 0). This observation could imply that this primary tumor is better supplied with blood (option A) and thus grows faster than the other tumors did.

### The absolute number of cancer cells of a tumor is difficult to assess but was not relevant for our simulation of the metastatic spread

The total of number of cancer cells of the primary tumor is calculated from its volume. However, the tumor consists of malignant cells, stroma cells, and blood vessels. Therefore, the absolute number of cancer cells in the primary tumor can be assumed to be lower than the estimate. This composition of the primary tumor indicates that our conclusions are still generally valid but that our estimated number of engrafting cells might be too high.

The colonization rate *β*(*x*) (Eq (2)) was used to compute the time between the extravasation of malignant cells from the primary tumor in relation to the number of cells in the primary tumor. It could be argued that this calculation is inaccurate as there are fewer living cancer cells than the estimate because of the necrotic core. However, the blood vessels of the primary tumor are located mostly at the margin of the tumor to the surrounding tissue, which is reflected by the fractal dimension of 2 or less. The error resulting from the necrotic cells is therefore negligible as the cells in the core of the primary tumor are not important for the calculated spreading behavior.

### Clinical implications

Our findings indicate that both RT and ChT destroy tumor cells and alter the blood vessels supplying the tumor. Usually tumor cells themselves are in the focus of modern targeted therapies. However, it is well known that also the surrounding stromal tissue and the tumor vasculature play important roles for local tumor growth, metastasis formation, and therapy response. By indicating that Cisplatin therapy and hypofractionated radiotherapy change the tumor vasculature, and that these effects have still been detectable 15 and 11 days post-treatment, our findings raise the question to which extent these alterations are relevant to the effect of Cisplatin therapy and hypofractionated radiotherapy and how they might influence the effect of any subsequent treatment. Therefore, further research is required to assess the importance of vascular changes to the interaction of different treatment modalities and the implications for optimizing combination therapy schemes in order to reduce the numbers of CTCs and DTCs and to improve clinical outcome.

## Conclusion

We have successfully applied a combined modeling and experimental approach to study the spread of malignant cells in a xenograft mouse model. Based on experimental data and the analysis with our simulation model CaTSiT, we deduce that chemo- and radiotherapy modify the geometry of the blood vessel tree of the primary tumor. We have shown that this approach is not only useful for developing a better understanding of the mechanisms of the spread of malignant cells but can also stimulate new experimental research. Besides these important findings this study can serve as an example in which experimental and computational approaches work synergistically to produce important results in cancer research and to further the development of next-generation therapies.

## Supporting information

S1 Text Supporting Methods. (DOCX)

S1 Fig The dependency of the blood vessel densities on the segmentation threshold. The blood vessel densities of the control group (black dots), the Cisplatin group (light grey crosses) and the radiotherapy group (dark grey diamonds) are shown as functions of the post processing threshold (A). The inset shows the same results for the three different treatment groups as Fig 6 does, but with a threshold of 17.7 μm^2^ instead 10.3 μm^2^. Both the inset to this figure and Fig 6 are created using the toolbox [22]. An asterisk indicates a significance level of p<0.05, two asterisks show a significance level of p<0.01. Dark grey boxes indicate one standard deviation of the vessel density. The light grey boxes indicate the 95% confidence interval of the arithmetic mean value. For crosschecking, the same region growing-based algorithm has also been applied to additional sections using a different staining method, namely double labeling immunohistochemistry with the blood vessel marked by DAB (brown) instead of Permanent Red (B). To the same data, also another, k-means based algorithm (cf. steps B1-B9 in the S1 Text) has been applied to give yet a very similar result (C).
(TIF)

S2 Fig Scheme of growth and spreading procedure. Panel A shows an untreated and Panel B a treated (e.g. chemotherapy) simulation procedure.
(TIF)

S3 Fig Impact of different fractal dimensions (FD) on the number of DTCs (y-axis) depending on the size on the primary tumor (x-axis). One sample mouse was simulated with three different fractal dimensions: FD = 1.5 (red line), FD = 1.75 (blue line) and with FD = 2.0 (black line). The graph shows that the fractal dimension is an important parameter to determine an exact result.
(TIF)

S4 Fig Spheroidal fit of a typical sample from the control group. The diagram shows the radius r in cm of the primary tumor sphere at different days of measurement.
(TIF)

S5 Fig Individual linear regression fit of the xenografted mouse tumor data (control group) for each mouse. In order to determine the number of engrafted tumor cells at day 0 (*N*_0_) and the growth rate constant a of the primary tumor, linear regressions were performed. Panel A shows the individual fit for each mouse. The left y-axis shows the number of cells of the primary tumor and the right y-axis shows the volume of the primary tumor. Panel B shows the determined parameters.
(TIF)

S6 FigIndividual linear regression fit of the xenografted mouse tumor data (chemotherapy group) for each mouse.Each diagram shows only the data points which were not affected by chemotherapy. In order to determine the number of engrafted tumor cells at day 0 ($N_{0}^{i}$) we used the previously determined arithmetic mean growth rate constant $\bar{a}$ from the control group (ref. section F in the S1 Text) for linear regression. Panel A shows the individual fit for each mouse and Panel B the determined parameters.(TIF)

S7 FigIndividual linear regression fit of the xenografted mouse tumor data (radiotherapy group) for each mouse.Each diagram shows the data points which were not affected by radiotherapy only. In order to determine the number of engrafted tumor cells at day 0 ($N_{0}^{i}$) we used the previously determined arithmetic mean growth rate constant $\bar{a}$ from the control group (see section F in the S1 Text) for linear regression. Panel A shows the individual fit for each mouse and Panel B the determined parameters.(TIF)

S1 Table Determined and used parameter values for simulation setup. (TIF)

S2 Table Parameters and results of the control group (untreated). The table is divided into three subgroups: (1) Experimental data, (2) Linear regression data and (3) Simulation data. (1) These data represent the parameters which were measured by PCR or determined by weigh. (2) These data were determined by the previously described linear regression approach. In (3), parameters show all values for simulations in this group which were computed by various simulation runs. The last two columns in this table show the simulation results from two different methods to determine the number of disseminated tumor cells in the lung. One with the fractal dimension by DTC (*δ*_*DTC*_) and one with the fractal dimension by the number of circulating tumor cells (*δ*_*CTC*_).
(TIF)

S3 Table Values and parameter of the chemotherapy group. The table is divided into three subgroups: (1) Experimental data, (2) Linear regression data and (3) Simulation data. (1) These data represents the parameters which were measured by PCR or determined by weigh. (2) These data were determined by the previously described linear regression approach and the mean growth rate constant from the control group. In (3), parameters show all values for simulations in this group which were computed by various simulation runs.
The values for the size of the primary tumor before the treatment was applied (*PT*_*treat start*_ column) were computed by multiplying the tumor volume by 10^9^.
In the “*DTC*_*Sim*_ after change of δ” column the error was calculated by simulating each mouse with all parameters with its determined value from regression, but in every complete simulation run of each mouse only one parameter differs to its maximum or minimum with respect to the parameters standard derivation. Finally, all possible results were computed following propagation of error.
The values in the column “Chosen *δ* after treatment” are the fractal dimension values which were set one day after the therapy. The calculated values of the fractal dimension *δ* indicate that chemotherapy affects the blood vessels’ geometry. A fractal dimension of 0 implies a complete destruction of the blood vessel geometry.
(TIF)

S4 Table Values and parameter of the radiation group. The table is divided into three subgroups: (1) Experimental data, (2) Linear regression data and (3) Simulation data. (1) These data represent the parameters which were measured by PCR or determined by weigh. (2) These data were determined by the previously described linear regression approach and the mean growth rate constant from the control group. In (3), parameters show all values for simulations in this group which were computed by various simulation runs.
The values for the size of the primary tumor before the treatment was applied (*PT*_*treat start*_ column) were computed by multiplying the tumor volume by 10^9^.
(TIF)
