# Supplementary material for: Radiotherapy and chemotherapy change vessel tree geometry and metastatic spread in a small cell lung cancer xenograft mouse tumor model
Source: PLoS One. 2017 Nov 6;12(11):e0187144. doi: 10.1371/journal.pone.0187144 (PMC5673169; doi:10.1371/journal.pone.0187144)
Supplement: S1 Text — (DOCX) [file pone.0187144.s001.docx]

Supporting Methods

**Radiotherapy and chemotherapy change blood vessel geometry and metastatic spread in a small cell lung cancer xenograft mouse tumor model**

Thorsten Frenzel^1,3 ¶^, Bertin Hoffmann^2 ¶^, Rüdiger Schmitz^1 ¶^, Anja Bethge^2^, Udo Schumacher^1^, Gero Wedemann^2 *^

^1^ Center for Experimental Medicine, Department of Anatomy and Experimental Morphology, University Cancer Center, University Medical Center Hamburg-Eppendorf, Martinistraße 52, 20246 Hamburg, Germany

^2^ Competence Center Bioinformatics, Institute for Applied Computer Science, University of Applied Sciences Stralsund, Zur Schwedenschanze 15, 18435 Stralsund, Germany

^3^ University Hospital Hamburg-Eppendorf, Ambulatory Center, Department for Radiation Oncology, Martinistraße 52, 20246 Hamburg, Germany

^*^ Corresponding author

^¶^ These authors contributed equally to this work

1. Immunohistochemistry for detecting endothelial cells

Paraffin-embedded primary tumors were subjected to conventional immunohistochemistry for specifically detecting endothelial cells and to double labeling immunohistochemistry for simultaneously marking endothelial cells and γH2AX foci.

After rehydration by xylene and graded alcohols, sections were pretreated with pH 9 Tris/EDTA buffer (Dako North America Inc., Carpinteria, USA, catalogue number S2367) at 121°C for 10 minutes.

Protocol i.: For conventional immunohistochemical staining of endothelial cells, primary anti-CD31 monoclonal antibody (Abcam, Cambridge, UK, catalogue number ab28364) was diluted 1:50 and incubated at room temperature for one hour. Secondary anti-CD31 antibody binding site were labeled by an avidin-biotin alkaline phosphatase complex (ABC-AP kit, Vector Laboratories Inc., Burlingame, USA, catalogue number AK-5000) and Permanent Red (Dako North America Inc., Carpinteria, USA, catalogue number K0640). Every incubation step was followed by two 5 minute washings in TBS-T and one 5 minute washing in TBS.

Protocol ii.: For double labeling immunohistochemistry, primary anti-CD31 monoclonal antibody was diluted 1:50 and incubated at room temperature for one hour (just like in the conventional immunohistochemistry protocol described above). Primary anti-histone H2AX monoclonal antibody (Abcam, Cambridge, UK, catalogue number ab81299) was diluted 1:300 and incubated at 4°C overnight. As above, all incubation steps were followed by two washes in TBS-T and one wash in TBS (5 minutes each). Anti-CD31 antibody binding sites were detected through an avidin-biotin horseradish peroxidase complex (ABC HRP kit, Vector Laboratories Inc., Burlingame, USA, catalogue number AK-5000) and 3,3′-diaminobenzidine tetrahydrochloride hydrate (DAB; Sigma-Aldrich GmbH, Munich, Germany) as the substrate. Anti-histone H2AX antibody binding sites were detected using an avidin-biotin alkaline phosphatase complex (ABC-AP kit, Vector Laboratories Inc., Burlingame, USA, catalogue number PK-6100) and Permanent Red (Dako North America Inc., Carpinteria, USA, catalogue number K0640).

Finally, sections were counterstained with hematoxylin, dehydrated and mounted.

1. Algorithms for Blood Vessel Segmentation

Automatic detection of blood vessels in the histological sections is achieved through a region growing-based segmentation scheme in {L,a,b} color space. The algorithm goes as follows:

1. Transform image to {L,a,b} color space
2. Seeding:
   1. Ignore all pixels which have an L value above a given threshold (L=200)
   2. In {a,b} subspace, compute the minimum distance of each pixel to (190|120) or (165|120), which corresponds to purple red and reddish colors (for permanent red staining)
   3. As the seed for subsequent region growing, take all pixels which are no further away from this point than 12 pixels (Euclidean distance in {a,b} subspace).
3. Region growing:
   1. In the three-pixel wide adjacency of the seed, exclude all pixels which have an L value higher than L=220 (i.e. relax the condition 2a. near seeding pixels)
   2. For each remaining pixel, compute the distances to (190|120) and (165|120) and take the minimum value.
   3. Take all pixels with a minimum distance value of no higher than 18 pixels as candidate pixels for the blood vessels (i.e. also relax the condition 2c near the seed).
4. The result is saved as a “vessel matrix” of the size of the original image with the candidate vessel pixels set to the 1 and all others set to 0
5. Find connected components in the vessel matrix and calculate their sizes.
6. Discard all connected components, which are smaller than a given post processing threshold, by setting their pixels to zero (see details below)
7. The resulting matrices are masks marking all pixels belonging to the blood vessels by 1 and marking all other pixels by 0.

This algorithm has been applied to all histological sections in which endothelial cells had been stained red. Note that for some sections, the threshold has been adjusted manually by hand in order to account for weaker or stronger vessel and background staining (see below).

For comparison and cross-checking, a second segmentation algorithm based on k-means clustering as been applied to certain slides:

1. Transform image to {L,a,b} color space
2. Ignore all pixels which have an L value above a given threshold (L=210) and project remaining pixels onto {a,b}-subspace
3. Run k-means clustering with $N_{rep}=8$ repetitions and $k=8$ cluster centers
4. For all cluster centers found by k-means, compute the Euclidean distance in {a,b}-space to the initial values chosen for the vessel color
5. Take those components, which are closest to the initial centers (hence minimize Euclidean distance), as vessel pixels
6. Compute a “vessel matrix” of the size of the original image with only the vessel pixels set to the digit 1 and all others set to 0 (as for the above algorithm).
7. Find connected components in the vessel matrix and calculate their sizes.
8. Discard all connected components, which are smaller than a given post-processing threshold, by setting their pixels to zero (as before).
9. The resulting matrices are masks marking all pixels belonging to the blood vessels by 1 and marking all other pixels by 0 (as before).

Color value-based seeding (step A2) as well as assigning individual pixels to color-space clusters (B5) are only dependent on single, individual pixel values, which makes them prone to noise. There will always be some objects falsely classified as (candidate) vessel pixels. However, most of these object will be smaller than actual blood vessels. Therefore, noise can effectively be reduced by imposing a threshold on the minimum size of any candidate blood vessel in order to be considered as an actual blood vessel (steps A5-A6 and B7-B8). The elimination of false positive pixels is achieved by the following procedure: from the binary matrix indicating all candidate pixels for belonging to a blood vessel by the digit one, sizes of all connected components of candidate pixels are calculated (step A5 and B7, respectively). Each connected component having a size smaller than a specified threshold is dumped (step A6 and B7). The remainder is an image in which all blood vessels are indicated through the digit one. The post-processing threshold employed in step A6 (B8) has to be chosen such that noise is suppressed as much as possible whilst blood vessels down to the smallest possible are being retained.

The data presented in the results section derives from post-processing the color-segmented data with a threshold corresponding to an area of 10.3 µm^2^ (corresponding to a diameter of 3.6 µm for a circular vessel cross section). Clearly, calculating blood vessels and computing blood vessel densities will very much depend on the choice of the threshold. (The same restrictions applies to an implicit threshold in manual counting). In order to rule out the influence of the threshold on the results derived from comparing the blood vessel densities between different groups, we have computed the blood vessel densities in each sample using multiple threshold values from 3.5 µm^2^ to 170 µm^2^. S1 Fig shows the dependency of the blood vessel densities on the threshold for the control group (black dots), the Cisplatin group (light grey crosses) and the radiotherapy group (dark grey diamonds). One can infer that the relative position of the different curves remains the same for a wide range of possible post-processing thresholds. This statement may be applied to a wide range of threshold values except very low (<< 10 µm^2^) and very high threshold values (approaching 350 µm^2^ and higher). For these values, the results are dominated by noise and by very big blood vessels, respectively. The latter are rare and in any case irrelevant for the microcirculation than the capillaries. Choosing a threshold of e.g. 17.3 µm^2^ (cf. S1 Fig, inset) yields the same results as presented in figure 6 (a) of the main text, where a threshold of 10.3 µm^2^ has been employed. For the former, the two-sample t-test rejects the null hypothesis at p = $0.010$ for cisplatin treatment and at p = $0.009$for RT. From the fact that these findings consistently extend to higher post-processing threshold values (28 µm^2^, 35 µm^2^, 70 µm^2^, …), which easily suppress noise, we conclude that a threshold value of 10.3 µm^2^ can already sufficiently suppress noise, whilst still retaining blood vessels of small diameters. We have checked the influence of the post-processing threshold for different combinations of segmentation algorithms and staining methods and found a similar behavior as described here for the region growing-based algorithm on conventional (single labeling) immunohistochemistry images. In these cases as well, a post-processing threshold in the range of 10 to 20 µm^2^ effectively reduces noise whilst preserving small vessels. Throughout the rest of the contribution paper and the entire main text and wherever not stated otherwise, a fixed post-processing threshold of 10.3 µm^2^ has been adopted.

All results presented in the main text of this paper are obtained by (at least) triple-determination of the vessel densities from specific Anti-CD31 immunohistochemical staining with Purple Red (S1, section A, protocol i.) through the region growing-based segmentation algorithm described in the steps A1-A9 of this section with a post-processing threshold of 10.3 µm^2^, with the following exceptions: the vessel densities for two out of ten tumors for the control group are obtained from only two histological sections each and the vessel density of one out of nine tumors from the ChT group is based on one histological sections alone. Bearing in mind that several of the tumors in the RT group had a smaller volume than those from the other two groups, their vessel densities are computed from four sections for three of them, and from five sections for another one. With the exceptions named here, the vessel densities for each individual tumor presented in the results section of this paper (cf. Fig. 6, grey dots) are obtained by triplicate, where each data point is the arithmetic mean of the vessel densities of three equally spaced histological sections (section-to-section spacing 100 µm).

This region growing-based segmentation algorithm has been applied to all histological slides with the blood vessel stained red outlined in steps A1-A7 and with the parameters given there. Afterwards, segmentations have been checked by inspection of the original sections and the recognized blood vessels. In order to account for variations in the strength of both vessel and background staining from section to section, the parameters have manually been adjusted in 28 out of 79 slices in total.

Besides visual inspection of the histological sections and the segmentation results, we have conducted the following crosschecks in order to rule out systematic errors in the staining or the segmentation protocol or the combination of them two: 1. We have applied the same segmentation algorithm to one additional slide per tumor which has been processed according to a well-established in-house protocol for double labeling immunohistochemistry, where the blood vessels are marked by DAB (brown) instead of Permanent Red (purple red), cf. S1 section A, protocol ii. 2. The slides stained by double labeling immunohistochemistry (S1, section A, protocol ii.) have, in addition, also been analyzed by the segmentation algorithm based on the well-known (but computationally more intensive) k-means clustering method (cf. steps B1-B9). Within both these settings, very similar differences between the treatment groups have been found, at very similar significance levels (cf. Fig S1, B and C).

1. Calculation of the Absolute Number of Disseminated Tumor Cells

With ALU-PCR, we obtained the numbers of lung DTCs per 60 ng template $\left( {PCR}_{template} \right)$ roughly corresponding to 10,000 murine cells. We calculated the absolute number of lung DTC per mouse regarding to their individual lung size as follows:

$$\frac{{PCR}_{template}}{correction factor \left( 500 \right)}=number of DTC per 60 ng DNA$$

$\frac{Number of DTC per 60 ng DNA*total lung size \left[ cells \right]}{10.000}=absolute number of DTC$

Example:

Right lung: 0.08g

Left lung: 0.11g

Total lung: 0.19g

$${PCR}_{template}=11.600$$

$\frac{11.600}{correction factor 500}=23.2 lung DTC per 10.000 murine cells$

We assumed 0.19 g of lung mass is equivalent to 190.000.000 cells. It is not important whether the assumption is correct, but we need this assumption to compute all mice with the same proportional factor.

| 23,2 cells | 10.000 cells |
| --- | --- |
| X | 190.000.000 cells |

X = 440800

1. Computer Model

The approach used in this manuscript for modeling tumor growth and the spreading behavior of metastases is based on the mathematical formalism originally developed by Iwata et al. [1]. The Cancer and Treatments Simulation Tool (CaTSiT) software was recently extended to include various treatment interventions [2]. In the following we describe the parts which were used in this publication. For an overview of the simulation model see S2 Fig.

## Compartments and events

CaTSiT is developed as a building kit. It is possible to create various scenarios with different kind of building blocks. The two main types of building blocks are compartments and events. A compartment is an object that can contain malignant cells such as the primary tumor, blood stream and metastasis. An event describes what happen in a compartment at a specific time. A local event affects only one compartment, whereas a global event can affect more than one compartment. For example, therapies can affect not only the primary tumor, but also metastasis.

Compartments can be modeled either continuous or discrete. In a continuous compartment, the growth of the compartment size is modelled by a growth function such as Gompertz or exponential and the spreading behavior are modelled by a colonization function which depends on the current size of the compartment. In a discrete compartment, all internal processes are modeled with events. The growth behavior of a discrete compartment is modeled by simulating each cell division, apoptosis or translocation into a different compartment. A set of possible events types can be defined for discrete compartments. Furthermore, each event has a probability of occurrence.

## Modeling tumor growth

The growth function $x(t)$ represents the number of cells in the tumor at time t, which is the solution of

|  | $\frac{dx}{dt}=g\left( x \right), x\left( 0 \right)=N_{0},$ | (1) |
| --- | --- | --- |

where $N_{0}$is the size of the primary tumor at$t=0$. Different functions can be chosen for the growth rate $g(x)$, such as linear, exponential, Gompertzian or power laws. Solving equation 1 with the determined exponential function for $g(x)$, the following equation represents the number of cells of the tumor at time t:

|  | $x\left( t \right)=N_{0}e^{at},$ | (2) |
| --- | --- | --- |

where $a$is the growth rate constant. In experimental setups, such as mouse models, the parameter $N_{0}$will depend on a specific value e.g. the number of injected tumor cells.

## Modeling metastatic spread

The number of cells that spread from the primary tumor per time unit is described by the colonization rate *β(x)*, as similarly shown by Iwata et al. [1]:

|  | $\beta\left( x \right)=mx^{\delta/3}$ | (3) |
| --- | --- | --- |

where$m$is the colonization coefficient and $\delta$ is the fractal dimension of blood vessels. The fractal dimension describes the blood vessel geometry and, thus, the nutrient supply of the primary tumor or metastases. S3 Fig show the difference between various fractal dimensions regarding to the number of cells which were spread from the primary tumor.

## Modeling Radiotherapy

The effect of external beam radiation therapy is computed with the help of the linear quadratic model [3]:

|  | $n_{s}= n_{b}e^{-\alpha D-\beta D^{2}}$ | (4) |
| --- | --- | --- |

where $n_{s}$is the number of surviving cells after the treatment and $n_{b}$is the number of cells in the tumor before treatment. Parameter $D$ is the radiation dose that is administered to the tumor during one treatment session. The parameter $\alpha$ describes the probability of creating a lethal double-strand break in the DNA and $\beta$ describes the probability of creating two single-strand breaks that lead to a lethal double-strand break. Thus, $\alpha$ and $\beta$ describe the radio-sensitivity of the radiated cells.

## Modeling Chemotherapy

The effect of the applied chemotherapy is modeled by the modified growth function [2,4]:

|  | $x_{C}\left( t \right)=N_{0}e^{\left( at+\frac{f\mu\left( e^{-\gamma t}-1 \right)}{\gamma} \right)}$. | (5) |
| --- | --- | --- |

where *a* is the growth rate constant and $N_{0}$ is the number of cells at time *t_c_*_,_ the start time of the chemotherapy. The parameter $f$ describes the fraction of cells that are in S phase in the cell cycle, which are the ones that will be affected by the chemotherapy. The drug-sensitivity parameter $\mu$describes the effectiveness of the chemotherapy and $\gamma$is the decay rate that specifies how fast the drug vanishes from the system. The decay rate can be calculated with the help of the drug half-life by$\gamma=\frac{ln(2)}{T_{1/2}}$.

## Piecewise-defined function

Because of the applied treatments during the experiment, the growth function of compartments must change at a specific time. Thus, a piecewise-defined growth function is implemented as a list and different function can be added. The corresponding function is selected in this list according to the time of its occurrence and describes the growth behavior of the compartment until a new function is selected.

## Simulation procedure

The different scenarios are configured with the help of XML (Extensible Markup Language) files. The primary tumor, the bloodstream and metastases in various organs are all modeled as compartments containing cancer cells. The primary tumor is described by continuous compartment, in which the growth and spreading behavior is defined by equation (2) and (3). The bloodstream is modelled as a discrete compartment. All events are stored in an event list. In each simulation step, the next event in time is selected and executed. As a result, an EXCEL file will be generated for each experimental data set which contains the current time in days, number of cells in the primary tumor, number of cells in the bloodstream, number of metastases, number of cells in all metastases and a size histogram of all metastases.

1. Choice of growth function

Tumor growth in many mouse models is better described by spheroidal growth than Gompertz growth especially at the beginning of the growth of the tumor [5]. Regarding to our experimental data, the radius $r_{t}$ of the primary tumor volume was calculated at each available time point to compute the growth rate constant *a* and the radius $r_{0}$ at the beginning of the experiment. The results achieved unrealistic findings about$r_{0}$. As an example, in S4 Fig, the results of a random mouse from the control group shows a negative parameter$r_{0.}$Therefore, we used an exponential growth function to describe the growth behavior.

1. Determining simulation parameters

## Determining growth parameters of the primary tumor

The measured volume size of the primary tumor was calculated by multiplying the number of cells by${10}^{9}$, based on the approximate size of one cell of 10x10x10 µm^3^. A linear regression including only the tumor data determined by palpation for each data set *i* resulted in values $a^{i}$ and$N_{o}^{i}$. Palpation tends to overestimate the size of the tumor because of the fur and skin of the animals and the weighed primary tumor (skin and fur removed) at necropsy is a more precise measurement. Thus, in the majority of cases, the final tumor size measured by weight was lower than that estimated by palpation in the control group. To compensate for this problem, we calculated an offset of all tumor data points that were determined by palpation by performing a linear regression. We computed the linear regression function value $x_{end}^{i,f}$, which were performed including only the palpation data, on the same day where the tumor data obtained by weight and calculated the difference by$\Delta x_{end}^{i}= x_{end}^{i,s}- x_{end}^{i,f}$, where$x_{end}^{i, s}$ is the value that was determined by weight. We performed this for all mice in the control group and calculated $\overline{{\Delta x}_{end}}$ as mean of all $\Delta x_{end}^{i}$. We added this offset to every data point that was determined by palpation. Finally, we performed a linear regression with these transformed data points (S5 Fig).

## Determining spread of malignant cells

To determine how spreading behavior corresponds to the geometry of the blood vessels of the primary tumor, we decided to vary δ for each mouse while assuming *m* to be constant. An analysis of both parameters was not possible with our experimental data. Furthermore, an adjustment of both parameters at the same time leads to an uncountable value constellation. As a reference, we chose the mouse with the highest primary tumor weight at the end of the experiment. The value for $\delta$ was fixed at 2 for this mouse as this primary tumor grew rapidly and had a superficial blood supply. With this parameter $\delta$ and the previously determined parameters (growth rate constant $a$and number of grown tumor cells$N_{0}$), the colonization coefficient$m$could be determined. We performed a simulation and adjusted $m$ until the simulated number of disseminated tumor cells (${DTC}_{Sim})$was almost identical ($\pm2.5\%)$ to the number of disseminated tumor cells as shown by Alu PCR (${DTC}_{PCR})$ [6]. The absolute number of lung DTCs for each mouse was calculated with respect to their individual lung volume size (see section C).

To determine the fractal dimension for the other mice, two approaches were compared:

(A) We simulated each mouse with the previously determined parameter $m$ and adjusted the fractal dimension $\delta_{DTC}$ until the number of ${DTC}_{Sim}$ reached the number of${DTC}_{PCR}$.

(B) We computed the fractal dimension $\delta_{CTC}$ based on the number of circulating tumor cells determined by Alu PCR (${CTC}_{PCR}$). Here, it is assumed that the number of CTCs is proportional to the colonization rate (Eq. (3)) with a proportionality constant$C$:

|  | $mx^{\delta_{DTC}/3}=C*{CTC}_{PCR}$. | (6) |
| --- | --- | --- |

The following procedure was used to determine $\delta_{CTC}:$

1. Compute the term $m{x_{end}}^{\delta_{DTC}/3}$ for the reference mouse. Parameter $x$is the number of cells of the weighed primary tumor at the end of the experiment.
2. Compute the constant of proportionality $C$between $m{x_{end}}^{\delta_{DTC}/3}$ and${CTC}_{PCR}$.
3. Use the transformation of equation (6) to compute $\delta$ for each mouse:

|  | $\delta_{{CTC}_{PCR}}= \frac{3LN\left( \frac{C*{CTC}_{PCR}}{m} \right)}{LN\left( x_{end} \right)}$ | (7) |
| --- | --- | --- |

Computer simulations for every mouse with $\delta_{DTC}$ and $\delta_{CTC}$were compared. Each simulation setup was computed about 100 times. After completion of each scenario the mean and standard deviation were computed.

## Determining parameters in treatment groups

For our computer model, we need to determine the parameters of the growth function for the primary tumor before the application of the therapy. The effects of chemo- and radiotherapy will be modeled separately with the help of therapy’s events (see section D). Therefore, we cannot use all data points which were measured in treated groups. Hence, to determine the engrafted tumor cells $N_{o}^{i}$ in the treated groups, we performed a linear regression including all data points before treatment (S6 and S7 Figs). We also add the previously determined offset from the control group. Consequently, the data points which have been altered by therapy’s can no longer falsify the linear regression result. We used the previously determined arithmetic mean growth rate constant $\bar{a}$ for the linear regression, because every mouse has the same cell line implanted. In cases where only one data point before treatment was measured, we directly computed the parameter $N_{o}^{i}$ with respect to the arithmetic mean growth rate constant $\bar{a}$ and this data point because a computerized linear regression is not possible with only one data point (S6 and S7 Figs).

### External beam radiation

No studies of the parameters $\alpha$ and $\beta$ for the radio-sensitivity of the radiated OH1 SCLC cell (Eq. (4) have been reported in the literature. Therefore, we used previously determined parameters from 15 other SCLC cell lines [7]. We calculated the average of all data to estimate the parameters for our simulations. The values of these parameters are $\alpha=0.434 {Gy}^{-1}$ and$\beta=0.079 {Gy}^{-2}$.

### Chemotherapy

The fraction of cells in S phase $f$ was estimated to be $10\%$. The exact value for *f* is actually not important for our simulation, since similar results were produced irrespective of whether this parameter was set in a range between 10% and 100%. The decay rate can be calculated with the help of the drug half-life (see section D). We assumed that the drug half-life of cisplatin is between $5$ and $50$ minutes [8]. In our simulation, we used a drug half-life of $27.5$ minutes and thus a drug decay rate $\gamma$of$36.60 \mathrm{day}^{-1}$ by calculating $\gamma=\frac{ln(2)}{T_{1/2}}$, which indicates how fast the drug vanishes from the system. The drug sensitivity parameter *μ* was assumed to be $0.5$as simulation in the ChT group showed that the variation of *μ* in the interval of 0.1 to$1$ had only minor effects ($<1\%$) on the number of disseminated tumor cells. As can be seen in the parameters, the chemotherapy is quickly out of the body without major effects on the primary tumor volume. Consequently, the chemotherapy that we applied was very weak.

1. **References (Supporting Methods**)

1. Iwata K, Kawasaki K, Shigesada N. A dynamical model for the growth and size distribution of multiple metastatic tumors. J Theor Biol. 2000;203: 177–186. doi:10.1006/jtbi.2000.1075

2. Bethge A, Schumacher U, Wedemann G. Simulation of metastatic progression using a computer model including chemotherapy and radiation therapy. J Biomed Inform. 2015;57: 74–87. doi:10.1016/j.jbi.2015.07.011

3. Bernhardt P, Speer TW. Modeling of the systemic cure with targeted radionuclide therapy. In: Speer TW, editor. Targeted Radionuclide Therapy. Lippincott Williams and Wilkins; 2010. pp. 263–281.

4. Wheldon TE. Models of tumour response to chemotherapy. Mathematical Models in Cancer Research. Bristol: Adam Hilger; 1988. pp. 157–179.

5. Yuhas JM, Li AP. Growth Fraction as the Major Determinant of Multicellular Tumor Spheroid Growth Rates. Cancer Res. 1978;38: 1528–1532.

6. Hoffmann B, Frenzel T, Schmitz R, Schumacher U, Wedemann G. Modelling growth of tumours and its spreading behaviour using mathematical functions. Cancer Bioinformatics. in Press.

7. Krarup M, Poulsen HS, Spang-Thomsen M. Cellular radiosensitivity of small-cell lung cancer cell lines. Int J Radiat Oncol Biol Phys. 1997;38: 191–196.

8. Siddik ZH, Jones M, Boxall FE, Harrap KR. Comparative distribution and excretion of carboplatin and cisplatin in mice. Cancer Chemother Pharmacol. 1988;21: 19–24.
