# Supplementary figures and images for: Radiotherapy and chemotherapy change vessel tree geometry and metastatic spread in a small cell lung cancer xenograft mouse tumor model
Source: PLoS One. 2017 Nov 6;12(11):e0187144. doi: 10.1371/journal.pone.0187144 (PMC5673169; doi:10.1371/journal.pone.0187144)

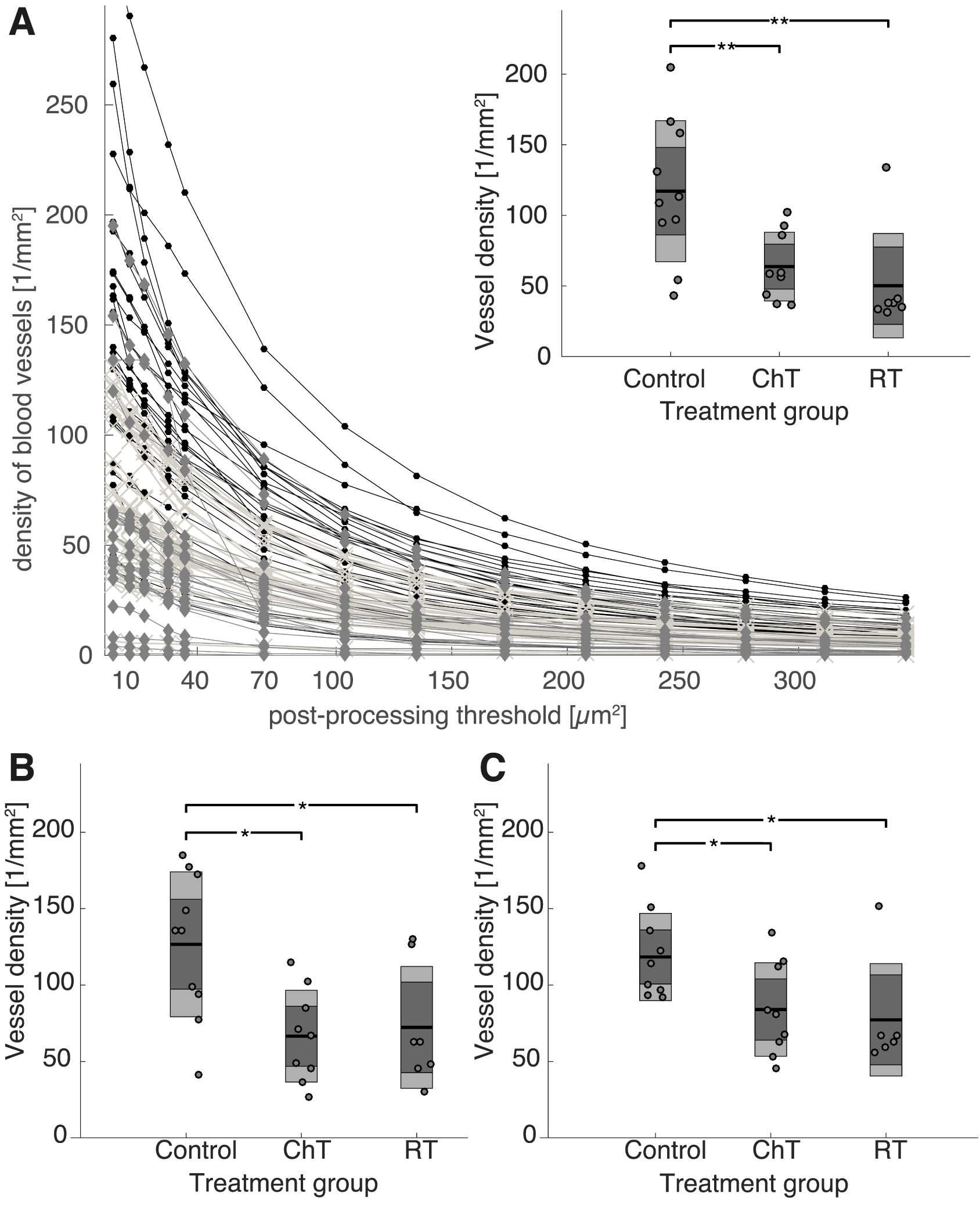

Supplement: S1 Fig — The blood vessel densities of the control group (black dots), the Cisplatin group (light grey crosses) and the radiotherapy group (dark grey diamonds) are shown as functions of the post processing threshold (A). The inset shows the same results for the three different treatment groups as Fig 6 does, but with a threshold of 17.7 μm2 instead 10.3 μm2. Both the inset to this figure and Fig 6 are created using the toolbox [22]. An asterisk indicates a significance level of p<0.05, two asterisks show a significance level of p<0.01. Dark grey boxes indicate one standard deviation of the vessel density. The light grey boxes indicate the 95% confidence interval of the arithmetic mean value. For crosschecking, the same region growing-based algorithm has also been applied to additional sections using a different staining method, namely double labeling immunohistochemistry with the blood vessel marked by DAB (brown) instead of Permanent Red (B). To the same data, also another, k-means based algorithm (cf. steps B1-B9 in the S1 Text) has been applied to give yet a very similar result (C). (TIF) [file pone.0187144.s002.tif]

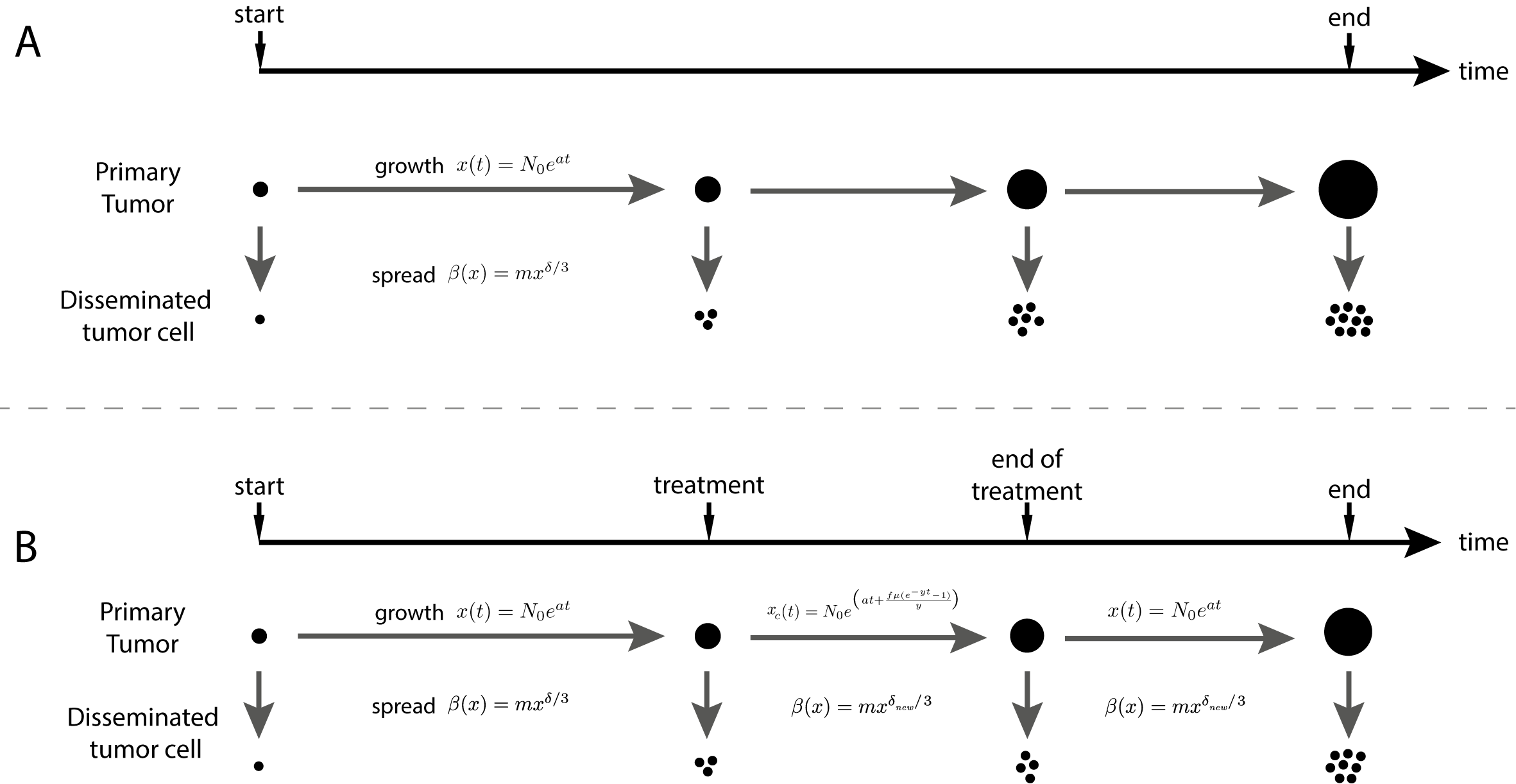

Supplement: S2 Fig — Panel A shows an untreated and Panel B a treated (e.g. chemotherapy) simulation procedure. (TIF) [file pone.0187144.s003.tif]

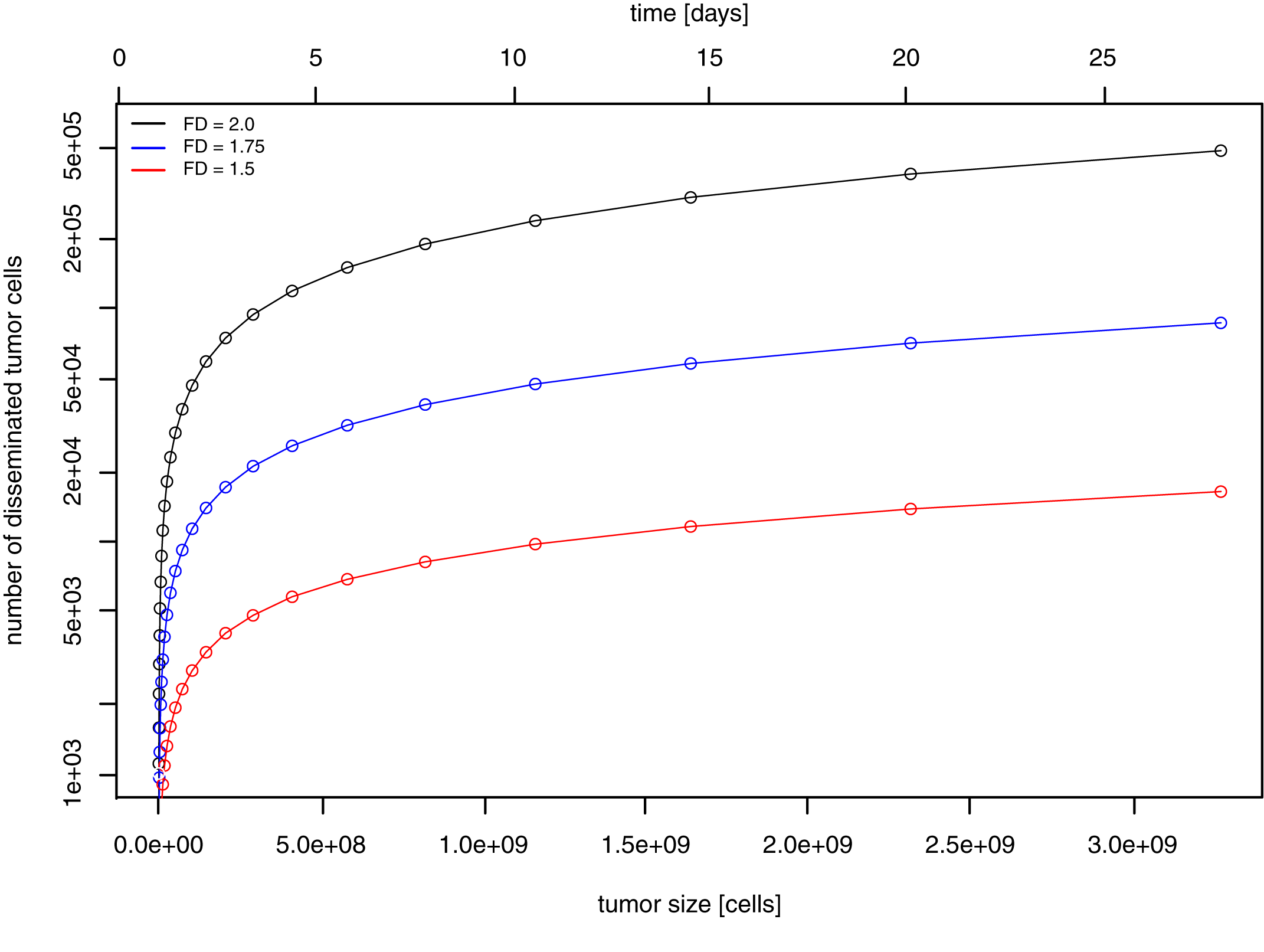

Supplement: S3 Fig — One sample mouse was simulated with three different fractal dimensions: FD = 1.5 (red line), FD = 1.75 (blue line) and with FD = 2.0 (black line). The graph shows that the fractal dimension is an important parameter to determine an exact result. (TIF) [file pone.0187144.s004.tif]

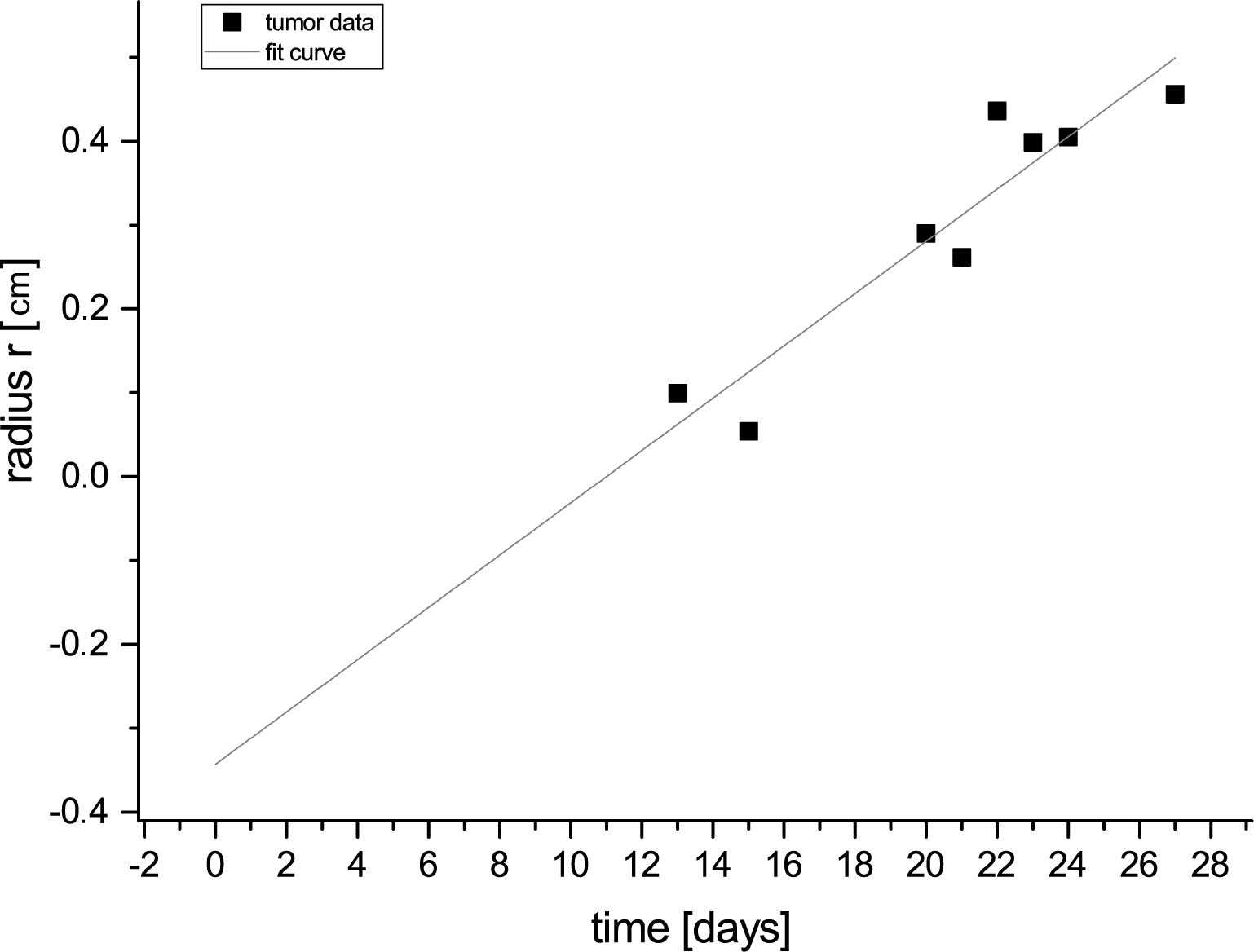

Supplement: S4 Fig — The diagram shows the radius r in cm of the primary tumor sphere at different days of measurement. (TIF) [file pone.0187144.s005.tif]

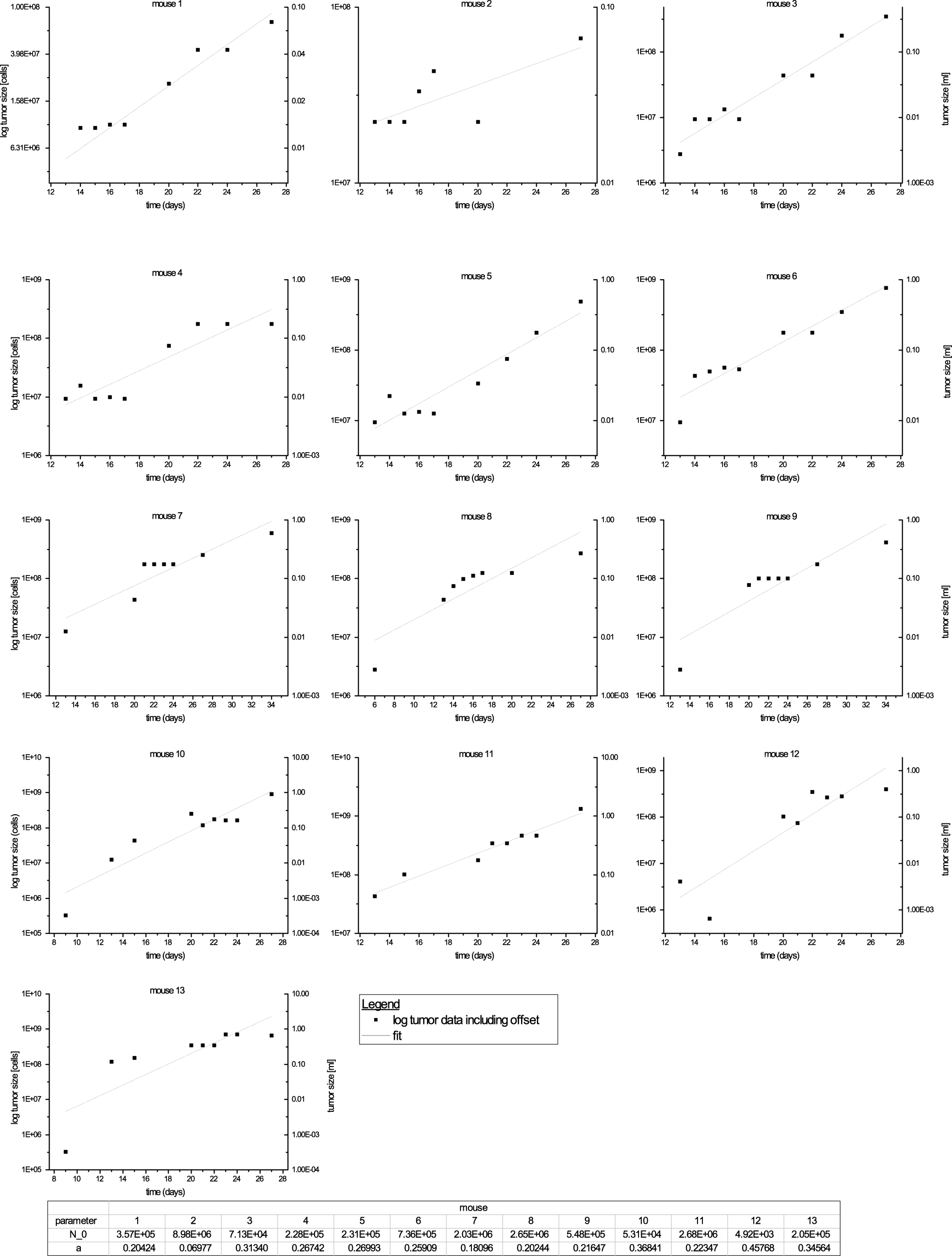

Supplement: S5 Fig — In order to determine the number of engrafted tumor cells at day 0 (N0) and the growth rate constant a of the primary tumor, linear regressions were performed. Panel A shows the individual fit for each mouse. The left y-axis shows the number of cells of the primary tumor and the right y-axis shows the volume of the primary tumor. Panel B shows the determined parameters. (TIF) [file pone.0187144.s006.tif]

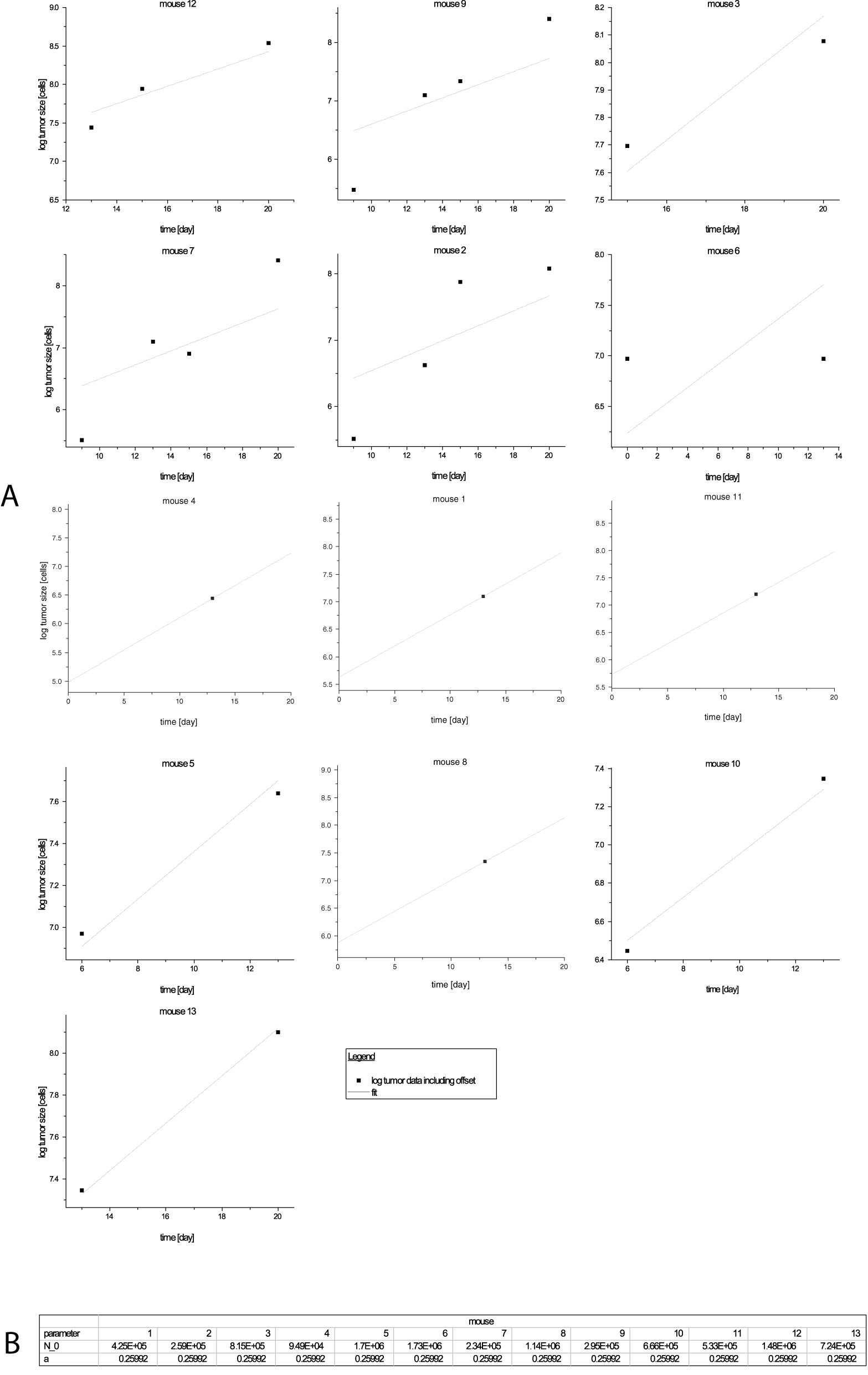

Supplement: S6 Fig — Each diagram shows only the data points which were not affected by chemotherapy. In order to determine the number of engrafted tumor cells at day 0 (N0i) we used the previously determined arithmetic mean growth rate constant a¯ from the control group (ref. section F in the S1 Text) for linear regression. Panel A shows the individual fit for each mouse and Panel B the determined parameters. (TIF) [file pone.0187144.s007.tif]

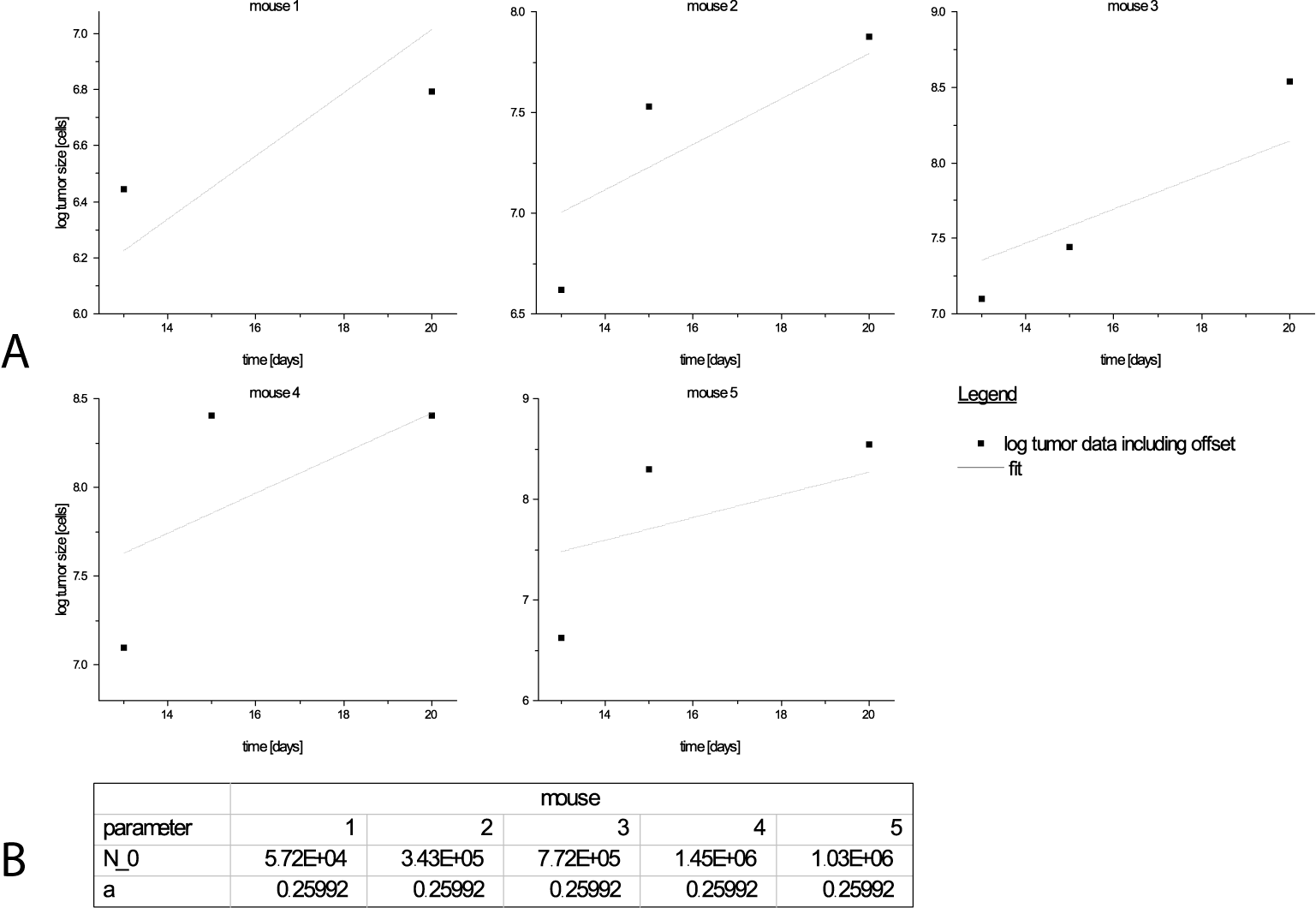

Supplement: S7 Fig — Each diagram shows the data points which were not affected by radiotherapy only. In order to determine the number of engrafted tumor cells at day 0 (N0i) we used the previously determined arithmetic mean growth rate constant a¯ from the control group (see section F in the S1 Text) for linear regression. Panel A shows the individual fit for each mouse and Panel B the determined parameters. (TIF) [file pone.0187144.s008.tif]

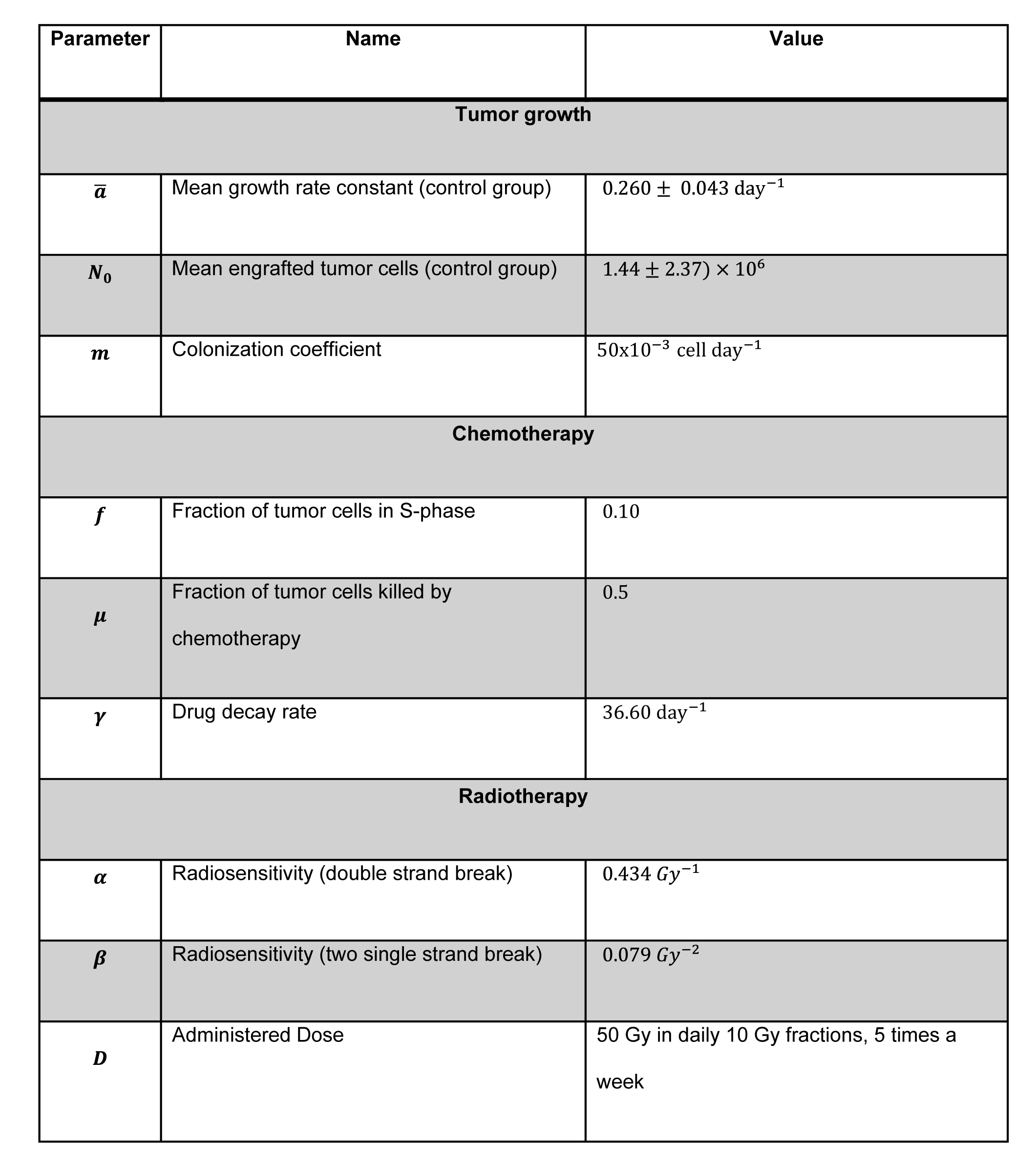

Supplement: S1 Table — (TIF) [file pone.0187144.s009.tif]

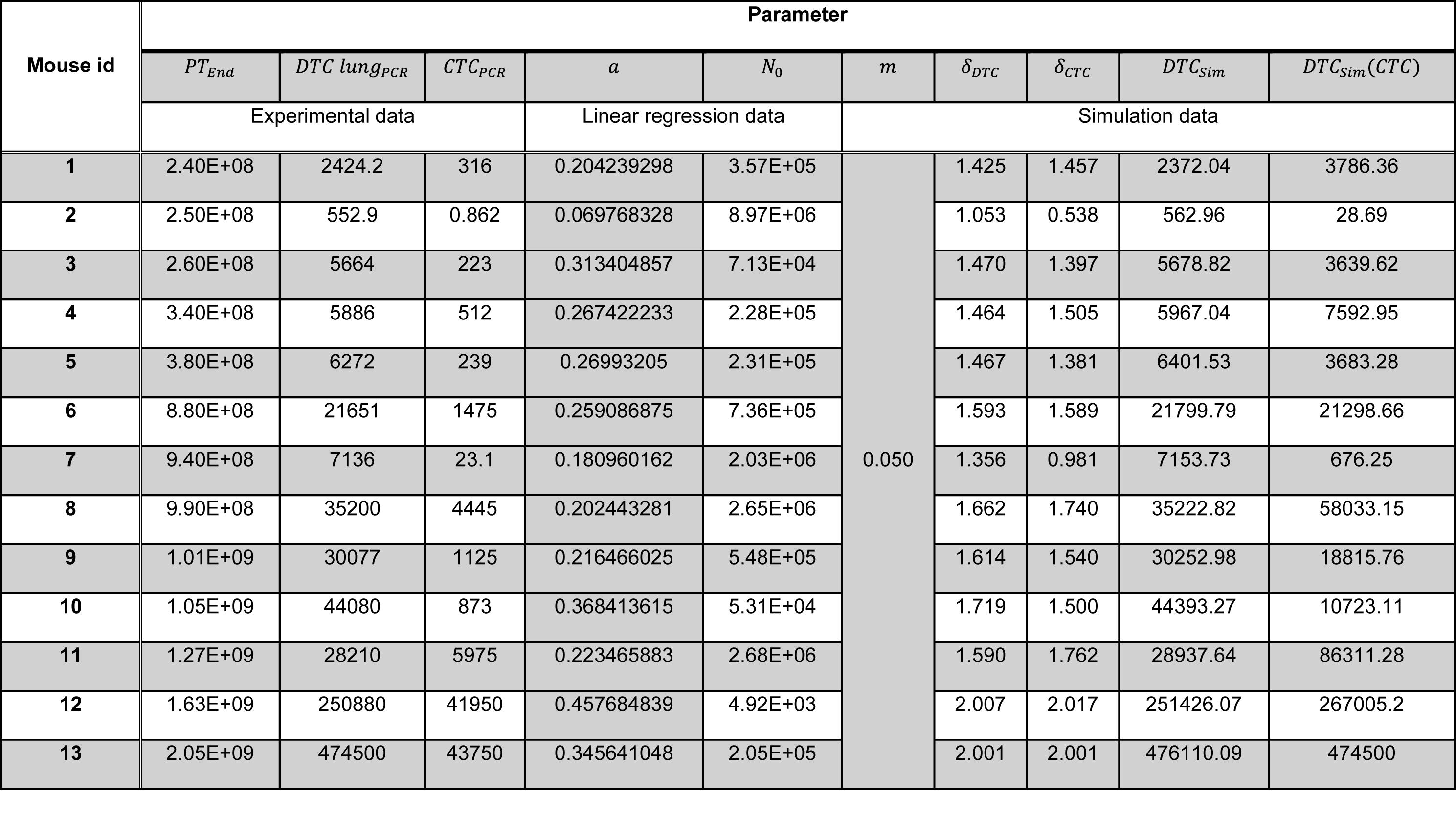

Supplement: S2 Table — The table is divided into three subgroups: (1) Experimental data, (2) Linear regression data and (3) Simulation data. (1) These data represent the parameters which were measured by PCR or determined by weigh. (2) These data were determined by the previously described linear regression approach. In (3), parameters show all values for simulations in this group which were computed by various simulation runs. The last two columns in this table show the simulation results from two different methods to determine the number of disseminated tumor cells in the lung. One with the fractal dimension by DTC (δDTC) and one with the fractal dimension by the number of circulating tumor cells (δCTC). (TIF) [file pone.0187144.s010.tif]

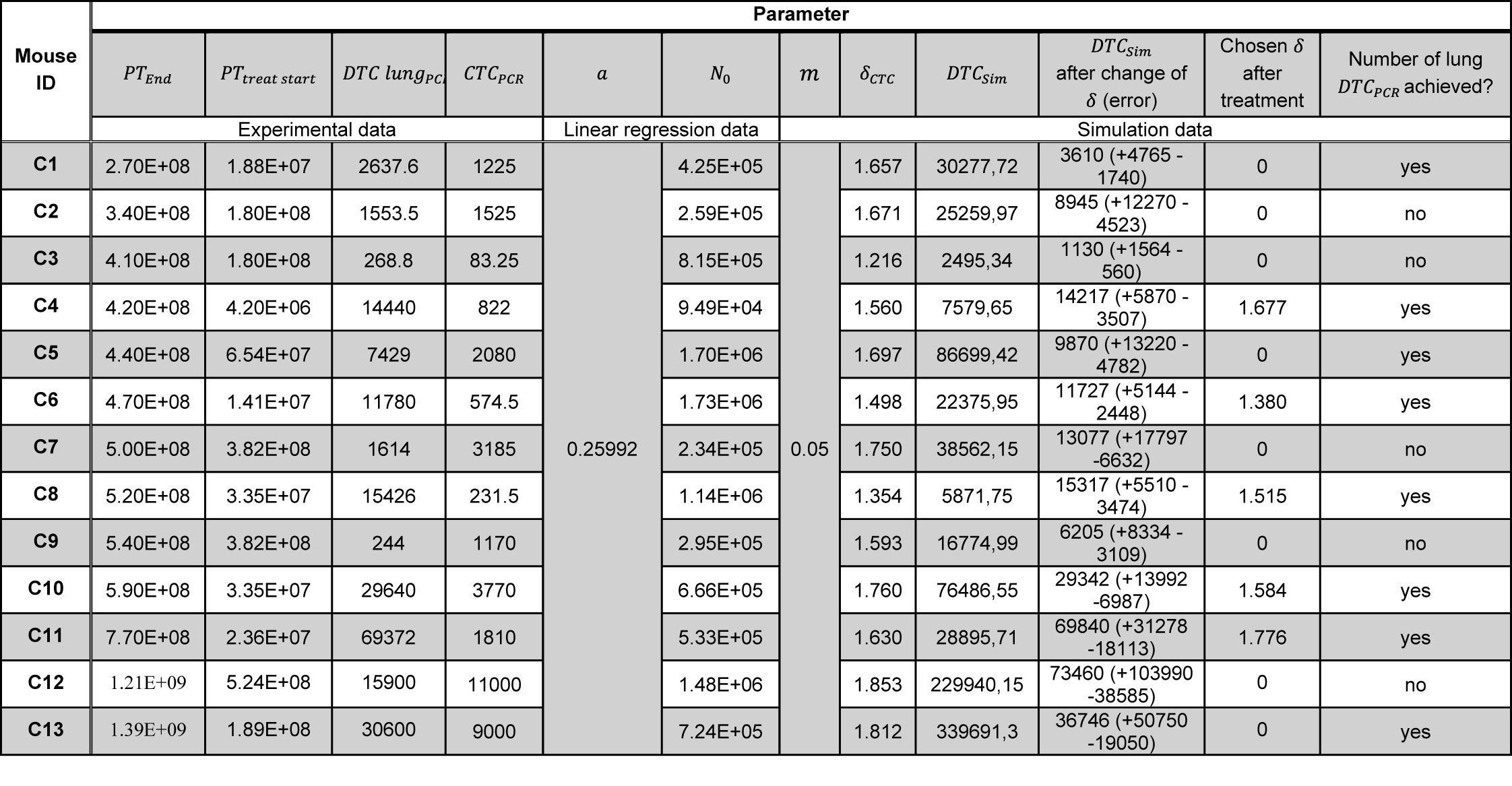

Supplement: S3 Table — The table is divided into three subgroups: (1) Experimental data, (2) Linear regression data and (3) Simulation data. (1) These data represents the parameters which were measured by PCR or determined by weigh. (2) These data were determined by the previously described linear regression approach and the mean growth rate constant from the control group. In (3), parameters show all values for simulations in this group which were computed by various simulation runs. The values for the size of the primary tumor before the treatment was applied (PTtreat start column) were computed by multiplying the tumor volume by 109. In the “DTCSim after change of δ” column the error was calculated by simulating each mouse with all parameters with its determined value from regression, but in every complete simulation run of each mouse only one parameter differs to its maximum or minimum with respect to the parameters standard derivation. Finally, all possible results were computed following propagation of error. The values in the column “Chosen δ after treatment” are the fractal dimension values which were set one day after the therapy. The calculated values of the fractal dimension δ indicate that chemotherapy affects the blood vessels’ geometry. A fractal dimension of 0 implies a complete destruction of the blood vessel geometry. (TIF) [file pone.0187144.s011.tif]

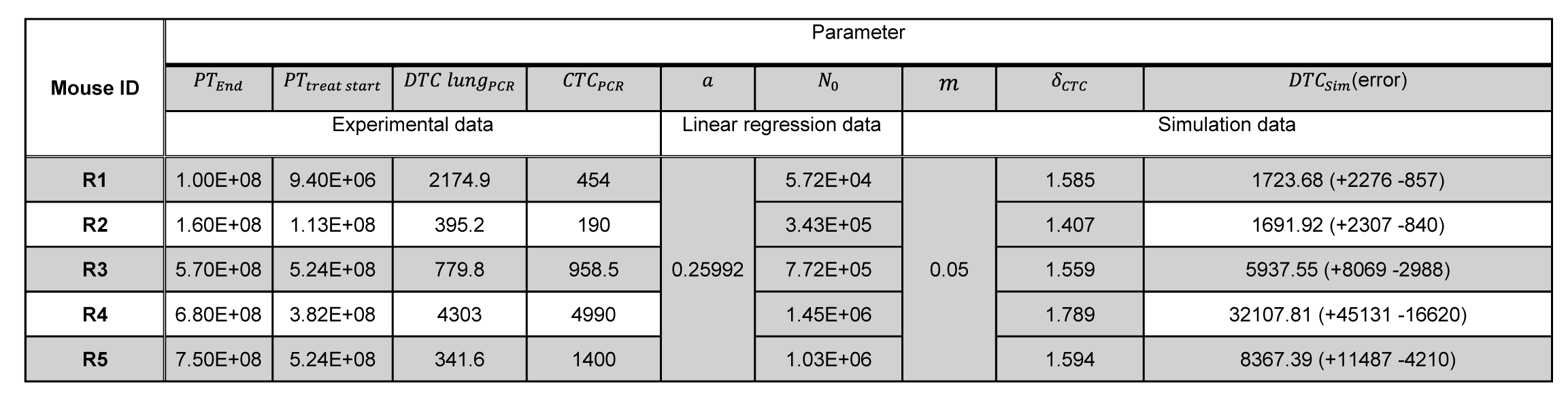

Supplement: S4 Table — The table is divided into three subgroups: (1) Experimental data, (2) Linear regression data and (3) Simulation data. (1) These data represent the parameters which were measured by PCR or determined by weigh. (2) These data were determined by the previously described linear regression approach and the mean growth rate constant from the control group. In (3), parameters show all values for simulations in this group which were computed by various simulation runs. The values for the size of the primary tumor before the treatment was applied (PTtreat start column) were computed by multiplying the tumor volume by 109. (TIF) [file pone.0187144.s012.tif]
